# Clipped histone H3 is integrated into nucleosomes of DNA replication genes in the human malaria parasite *Plasmodium falciparum*

**DOI:** 10.15252/embr.201846331

**Published:** 2019-03-04

**Authors:** Abril Marcela Herrera‐Solorio, Shruthi Sridhar Vembar, Cameron Ross MacPherson, Daniela Lozano‐Amado, Gabriela Romero Meza, Beatriz Xoconostle‐Cazares, Rafael Miyazawa Martins, Patty Chen, Miguel Vargas, Artur Scherf, Rosaura Hernández‐Rivas

**Affiliations:** ^1^ Departamento de Biomedicina Molecular Centro de Investigación y de Estudios Avanzados del Instituto Politécnico Nacional (IPN) Ciudad de Mexico México; ^2^ Unité Biologie des Interactions Hôte‐Parasite Département de Parasites et Insectes Vecteurs Institut Pasteur Paris France; ^3^ CNRS, ERL 9195 Paris France; ^4^ INSERM, Unit U1201 Paris France; ^5^ Departamento de Biotecnología y Bioingeniería Centro de Investigación y de Estudios Avanzados del Instituto Politécnico Nacional (IPN) Ciudad de México México; ^6^ Present address: Institute of Bioinformatics and Applied Biotechnology Bengaluru India

**Keywords:** epigenetics, histone H3 clipping, malaria, Chromatin, Epigenetics, Genomics & Functional Genomics, Microbiology, Virology & Host Pathogen Interaction

## Abstract

Post‐translational modifications of histone H3 N‐terminal tails are key epigenetic regulators of virulence gene expression and sexual commitment in the human malaria parasite *Plasmodium falciparum*. Here, we identify proteolytic clipping of the N‐terminal tail of nucleosome‐associated histone H3 at amino acid position 21 as a new chromatin modification. A cathepsin C‐like proteolytic clipping activity is observed in nuclear parasite extracts. Notably, an ectopically expressed version of clipped histone H3, PfH3p‐HA, is targeted to the nucleus and integrates into mononucleosomes. Furthermore, chromatin immunoprecipitation and next‐generation sequencing analysis identified PfH3p‐HA as being highly enriched in the upstream region of six genes that play a key role in DNA replication and repair: In these genes, PfH3p‐HA demarcates a specific 1.5 kb chromatin island adjacent to the open reading frame. Our results indicate that, in *P. falciparum*, the process of histone clipping may precede chromatin integration hinting at preferential targeting of pre‐assembled PfH3p‐containing nucleosomes to specific genomic regions. The discovery of a protease‐directed mode of chromatin organization in *P. falciparum* opens up new avenues to develop new anti‐malarials.

## Introduction

Malaria, a vector‐borne disease transmitted by female *Anopheles* mosquitoes, still kills a child every minute 1. The most severe form of human malaria is caused by the unicellular protozoan parasite *Plasmodium falciparum*, with clinical malaria symptoms manifesting during the 48‐h asexual blood‐stage cycle of the parasite. In the absence of efficient vaccines and the increasing spread of parasite drug resistance, treatment failures present a huge problem for malaria control and elimination. Over the past two decades, contributions to basic malaria research, particularly in the field of gene regulation, have identified new promising targets for anti‐malarial drug development. For example, several studies have demonstrated a central role for post‐translational modifications (PTMs) of histone N‐terminal tails in regulating key *P. falciparum* intra‐erythrocytic biology, antigenic variation, malaria pathogenesis, development of sexual stages that are transmitted to the vector, and virulence gene expression 2, 3, 4, 5, 6, 7. Accordingly, small molecules targeting these PTMs are in the pipeline for anti‐malarial development 8, 9, 10.

As observed in virtually all eukaryotes, the nucleosome is the basic unit of chromatin structure in *P. falciparum* and comprises an octamer of core histones, around which are wrapped 147 bp of DNA. *Plasmodium falciparum* has four canonical core histones H2A, H2B, H3, and H4, and four histone variants H2A.Z, H2Bv, H3.3, and CenH3 11. *Plasmodium falciparum* also has a rich complement of chromatin‐modifying proteins, and in fact, mass spectrometric analyses have shown that *Plasmodium* histones carry more than 60 PTMs, including acetylation, methylation, and phosphorylation 12, 13, 14. However, only a few have been studied in depth. Much of our knowledge about the role of PTMs in *P. falciparum* gene regulation comes from the investigation of the clonally variant expression of the *var* gene family 7, 15 and, more recently, the sexual commitment mechanism via variegated expression of a master regulator called AP2‐G, a transcription factor of the ApiAP2 family 16, 17.

In contrast to these reversible chemical modifications, it has recently emerged that proteolysis of histone tails is a type of irreversible PTM in eukaryotes, which can be resolved only by histone turnover or nucleosome remodeling. The consequences of histone tail processing include cell cycle progression, organismal development, viral infection, and aging 18, 19. For example, in mouse embryonic stem cells, it was shown that the clipping of the tail of histone H3 regulates cell differentiation 20. A second study identified an endopeptidase that cleaves the tail of histone H3 in *Saccharomyces cerevisiae* and showed that the processed form of histone H3 controls the induction of gene expression by potentially clearing repressive PTMs 21. Finally, a recent study demonstrated that mast cell lineage is governed by the tryptase‐mediated clipping of histone H3 and H2B tails 22. Thus, the biological outcome of cleavage of histone H3 differs in different organisms and cell types indicating that the clipped form is not just an intermediate of protein turnover.

In this work, we identified for the first time in a protozoan pathogen the clipping of the N‐terminal region of histone H3 at amino acid 21, deleting the N‐terminal tail from amino acids 1–21: This region is highly methylated and acetylated at positions lysine 4, lysine 9, lysine 14, and lysine 18, with particular marks being associated with transcriptional activation (H3K4me3 and H3K9ac) and others with regulation of variegated gene expression (H3K9me3), including of virulence genes involved in immune evasion and genes regulating sexual commitment 4, 5, 7, 15, 17, 23, 24. We also show that clipped histone H3 primarily integrates into chromatin regions upstream of six DNA replication gene loci hinting at the existence of a highly specific cellular targeting machinery for truncated histones. Overall, our data identify a novel epigenetic mechanism employed by *P. falciparum* that is linked to DNA metabolism.

## Results

### The N‐terminal region of histone H3 is clipped at amino acid position 21 in *Plasmodium falciparum* intra‐erythrocytic stages

**Figure 1:**
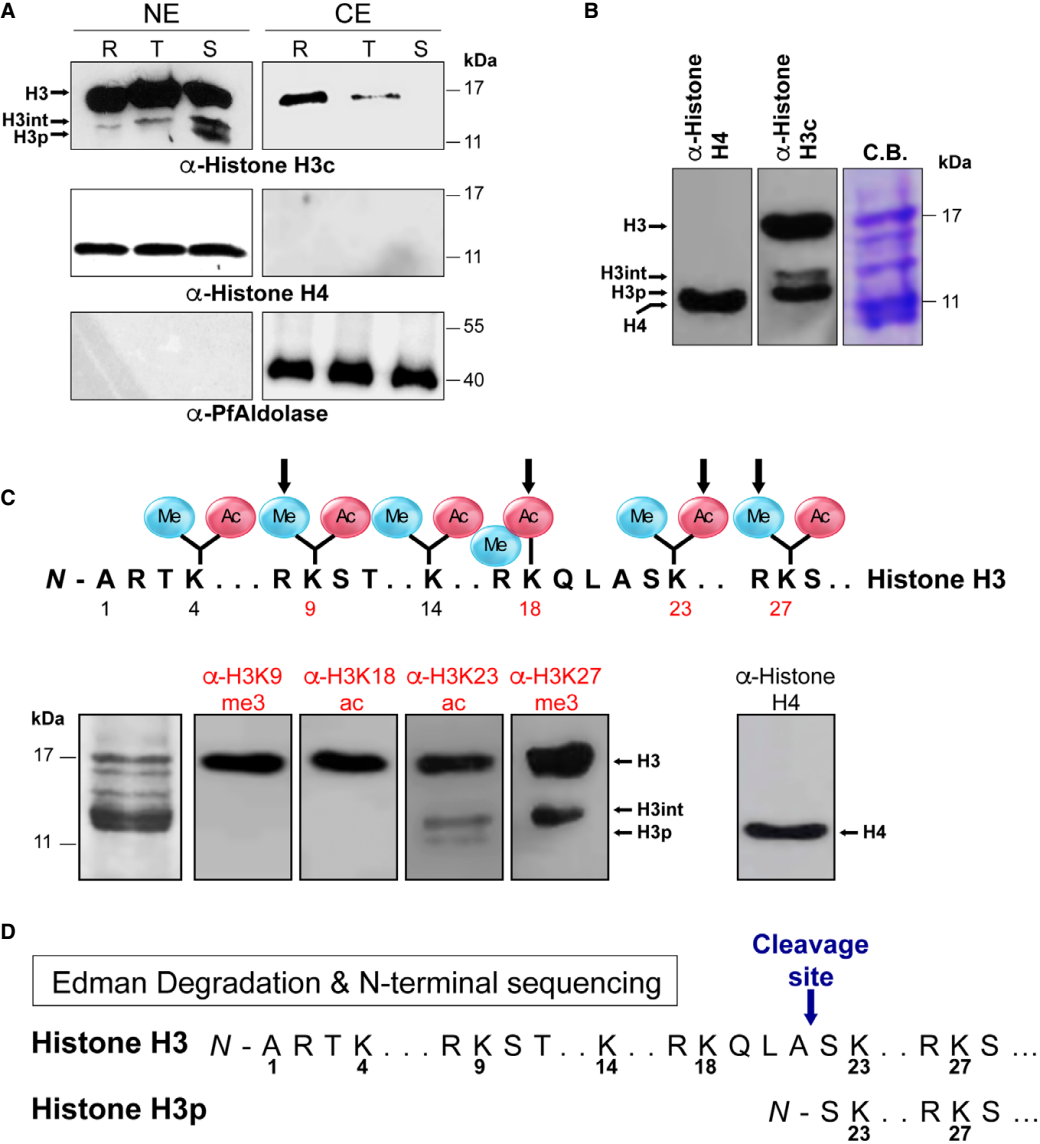
*Plasmodium falciparum* nucleosomal histone H3 is proteolytically processed in a stage‐specific manner between amino acids 21 and 22 Immunoblot analysis of nuclear and cytoplasmic extracts prepared from parasites synchronized at ring (R), trophozoite (T), and schizont (S) stages with anti‐histone H3 C‐terminus antibodies identifies three bands: full‐length histone H3, an intermediate form (H3‐int), and a fully processed form (H3p). Anti‐histone H4 and anti‐PfAldolase antibodies served as loading controls for the nuclear and cytoplasmic fraction, respectively.Immunoblot assays of mononucleosomes with anti‐histone H3 C‐terminus or anti‐histone H4 antibodies. The right panel shows a mononucleosome preparation separated on a denaturing polyacrylamide gel and stained with Coomassie Brilliant Blue (C.B.).Immunoblot analysis of a schizont‐stage histone preparation with antibodies against the indicated post‐translational modification (PTM) in the N‐terminal tail of histone H3 (upper panel).Results from the Edman degradation analysis of full‐length PfH3 or processed PfH3p; the arrow indicates the position of histone H3 clipping. Source data are available online for this figure.

To determine whether histone proteolysis occurs during *P. falciparum* intra‐erythrocytic development, we prepared nuclear and cytoplasmic extracts of 3D7 parasites synchronized at the ring [6–10 hours post‐invasion (hpi)], trophozoite (26–30 hpi), or schizont (36–40 hpi) stages, and analyzed them by immunoblotting with antibodies targeting the C‐terminus of histone H3 or histone H4 (Fig 1A). The resulting pattern consisted of a 17 kDa band corresponding to full‐length histone H3 (PfH3), a minor 14.5 kDa band, referred to here as the intermediate form PfH3int, and a 14 kDa terminally processed form PfH3p that was most prominent in schizonts (Fig 1A). In contrast, histone H4 migrated as a single band in all stages. Notably, we observed that the truncated forms of PfH3 are part of mononucleosomes prepared from the schizont stage (Fig 1B) and are not recognized by antibodies targeting the N‐terminus of histone H3 (Appendix Fig S1). These data indicate that N‐terminal processing of *P. falciparum* histone H3 to H3p occurs during blood‐stage development and that the processed form is incorporated into nucleosomes.

Next, to determine the position at which histone H3 truncation occurs, we recovered the 17 and 14 kDa bands from the polyacrylamide gel of Fig 1B and analyzed them by mass spectrometry. This analysis not only confirmed that the 14 kDa band was indeed a truncated form of histone H3 (Table EV1) and not the variant H3.3, but also identified previously known 12 and new histone PTMs in *P. falciparum* (mainly methylation of lysines and arginines; Table EV2). To further validate this finding, we probed schizont‐stage mononucleosomes using antibodies that recognize different N‐terminal histone H3 PTMs and found that PfH3p contained acetylated lysine 23 (H3K23ac) and tri‐methylated lysine 27 (H3K27me3) marks but not H3K9me3 or H3K18ac (Fig 1C). Together, these results predicted a cleavage site between amino acids 18 and 23. Finally, to identify the exact position of PfH3 cleavage, we analyzed PfH3p using Edman degradation. This analysis showed that the cleavage site is located between amino acids 21 and 22 (Fig 1D).

### The protease inhibitor profile of an *in vitro* histone H3 proteolysis assay predicts a cathepsin C‐like nuclear protease as being responsible for clipping

**Figure 2:**
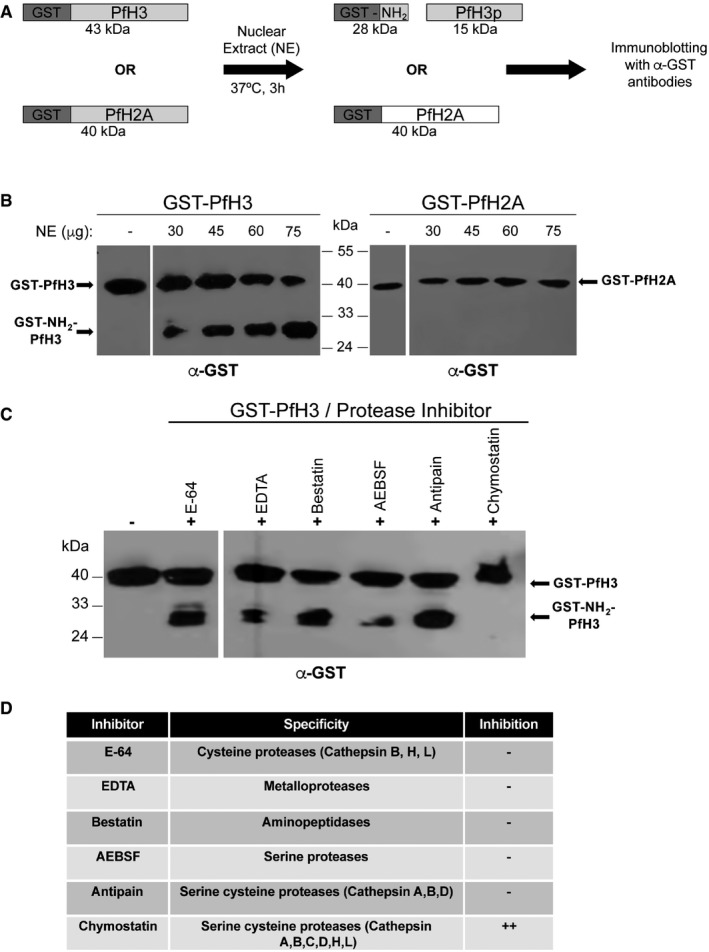
A cathepsin C‐type protease in schizont‐stage nuclear extracts may mediate *Plasmodium falciparum* histone H3 processing Schematic representation of the *in vitro* proteolysis assay developed in this study. N‐terminally GST‐tagged PfH3 was incubated with schizont‐stage nuclear extracts at 37°C for 3 h and analyzed by immunoblotting with anti‐GST antibodies. GST‐tagged PfH2A served as a control.GST‐PfH3 or GST‐PfH2A was incubated with increasing amounts of nuclear extracts, and the reactions were resolved using denaturing gel electrophoresis and analyzed by immunoblotting with anti‐GST antibodies.GST‐PfH3 was incubated with 25 μg of nuclear extracts and the indicated protease inhibitor. The resulting products were analyzed by denaturing gel electrophoresis and immunoblotting with anti‐GST antibodies. (−) indicates a reaction without nuclear extract and protease inhibitor.Target profile of the protease inhibitors used in panel (C). Source data are available online for this figure.

To identify the class of protease(s) involved in H3 processing, we developed an *in vitro* proteolysis assay using recombinant *P. falciparum* histone H3 fused to glutathione S‐transferase at the N‐terminus (GST‐PfH3) as substrate, and schizont‐stage nuclear extracts as the source of the proteolytic enzyme (Fig 2A); recombinant *P. falciparum* histone H2A tagged with GST (GST‐PfH2A) served as a negative control. As shown in Fig 2B, incubation of GST‐PfH3 (~ 43 kDa) with increasing amounts of nuclear extracts resulted in a truncated version GST‐NH_2_‐H3 (~ 28 kDa), which migrated faster than the full‐length protein on a polyacrylamide gel. In contrast, GST‐PfH2A was not processed (Fig 2B, right). Subsequently, to determine the nature of the PfH3 protease, we performed our *in vitro* proteolysis assay in the presence of chemical inhibitors targeting different protease families including cysteine proteases, metalloproteases, aminopeptidases, or serine–cysteine proteases (Fig 2C and D). Only chymostatin, an inhibitor of serine–cysteine proteases of the cathepsin A, B, C, D, H, and L family, blocked GST‐PfH3 processing (Fig 2C and D). However, because E64 (an inhibitor of cathepsin B, H, and L) and antipain (an inhibitor of cathepsin A, B, and D) did not abrogate GST‐PfH3 processing, we infer by elimination that a cathepsin C‐like protease found in the parasite nucleus may control the processing of histone H3 in intra‐erythrocytic stages. A similar prediction was made using the peptide cutter server (http://www.dmbr.ugent.be/prx/bioit2-public/SitePrediction/), which predict that the cleavage site of *P. falciparum* histone H3 occurs at leucine 21 and that this cleavage could be performed by cathepsin C.

### Ectopically expressed clipped H3 integrates into nuclear mononucleosomes

**Figure 3:**
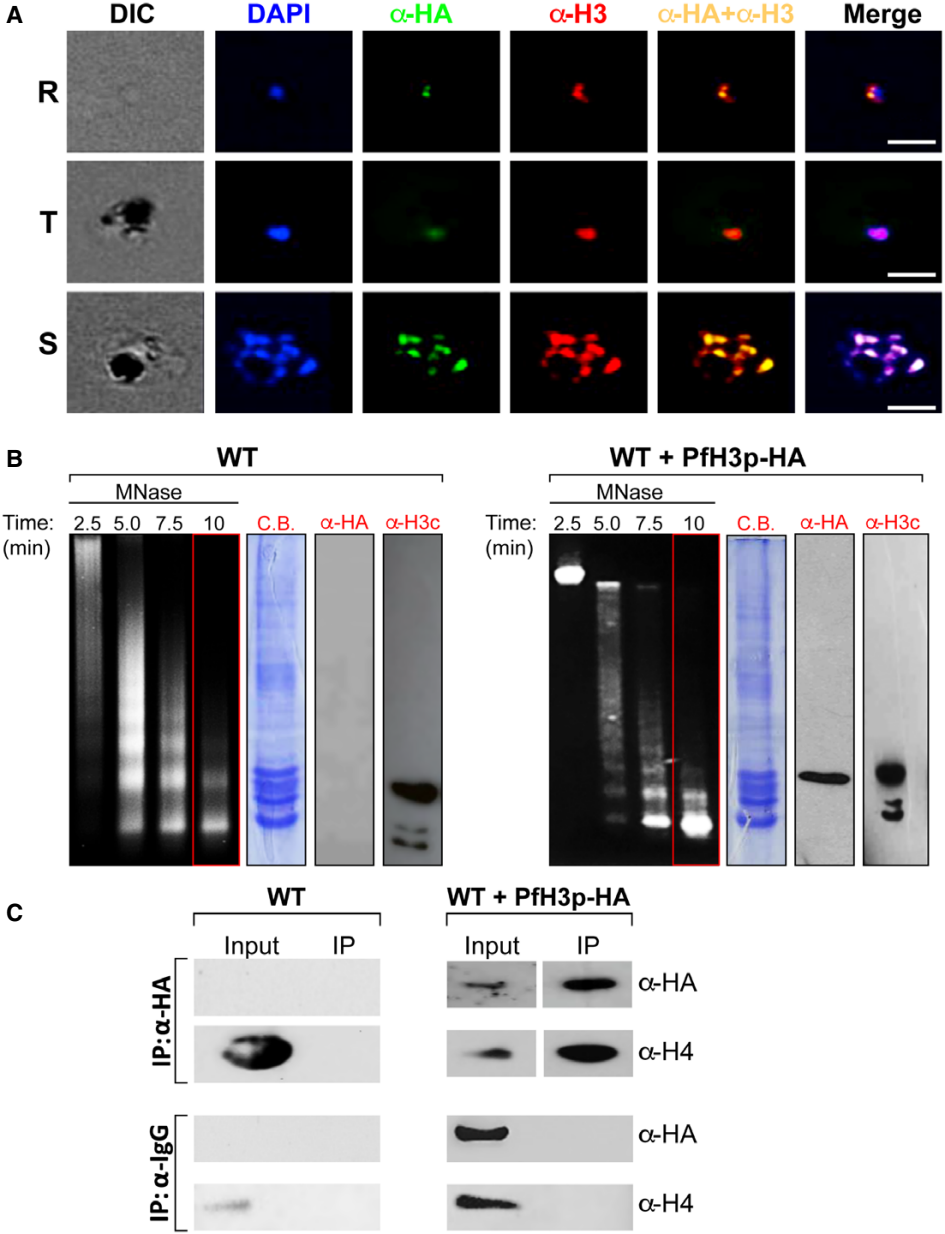
Ectopically expressed histone H3p localizes to the nucleus during parasite asexual development and incorporates into nucleosomes Indirect immunofluorescence assays were performed to determine the localization of ectopically expressed PfH3p‐HA in ring (R), trophozoite (T), and schizont (S) stages of *Plasmodium falciparum* asexual growth. PfH3p‐HA was detected using anti‐HA antibodies (green) and endogenous histone H3 with anti‐histone H3 N‐terminal antibodies (red). DAPI (blue) was used to stain the nucleus. Scale bar = 5 μm.Nuclei isolated from wild‐type (WT) or PfH3p‐HA‐expressing (WT + PfH3p‐HA) schizont‐stage parasites were treated with 4 U/ml of micrococcal nuclease (MNase) for the indicated amounts of time, the DNA purified and migrated on a 2% agarose gel, and stained with ethidium bromide. Mononucleosomes purified after 10 min of MNase treatment were separated using denaturing polyacrylamide gel electrophoresis and either stained with Coomassie Brilliant Blue (C.B.) or visualized by immunoblotting with anti‐HA (α‐HA) or anti‐C‐terminal histone H3 (α‐H3c) antibodies.Co‐immunoprecipitation (IP) experiments of purified mononucleosomes obtained from wild‐type (WT) or transfected (WT + PfH3p‐HA) schizont‐stage parasites were performed with either anti‐HA antibodies or mouse IgG. Immunoprecipitated products (right panel) were analyzed by immunoblotting using anti‐HA or anti‐histone H4 antibodies. Source data are available online for this figure.

We next investigated if histone H3 clipping occurs in *cis*‐, i.e., in the context of the nucleosome, or in *trans*‐, i.e., the clipped form of histone H3 could be integrated into nucleosomes by chromatin remodeling enzymes. To distinguish between these models, we expressed PfH3p ectopically from an episome with a C‐terminal triple‐HA tag under the control of the weak CRT promoter (see Material and Methods for more information) and confirmed PfH3p‐HA expression in our transgenic parasites (Appendix Fig S2). Thereafter, we performed immunofluorescence assays on fixed asexual blood‐stage parasites using anti‐HA antibodies and found that the PfH3p‐HA signal was restricted to the nucleus in all three developmental stages, the rings, trophozoites, and schizonts (Fig 3A). Furthermore, using purified mononucleosomes from wild‐type (WT) and transgenic (WT + PfH3p‐HA) parasites synchronized at the schizont stage, we found that PfH3p‐HA forms an integral part of the nucleosomes (Fig 3B). Importantly, the clipping of endogenous histone H3 is not lost in the transgenic parasites (Fig 3B, lanes labeled H3c). Lastly, immunoprecipitation experiments of purified mononucleosomes obtained from WT or WT + PfH3p‐HA schizont‐stage parasites with anti‐HA antibodies co‐immunoprecipitated histone H4 whereas the mouse IgG control did not (Fig 3C). These data indicate that ectopically expressed PfH3p‐HA interacts normally with canonical histones in the context of nucleosomes supporting a *trans‐* model for clipped histone H3 incorporation into *P. falciparum* chromatin.

### Clipped histone H3 is targeted to the 5′UTR of genes regulating DNA replication

Based on the above observation, we decided to use our transgenic parasite line to identify genomic regions that are targeted by clipped histone H3. Using anti‐HA antibodies, which immunoprecipitate PfH3p‐HA, and anti‐N‐terminal histone H3 antibodies, which immunoprecipitate histone H3 with an intact N‐terminal tail (referred to hereafter as PfH3n), we performed chromatin immunoprecipitation (ChIP) followed by next‐generation sequencing (NGS) (‐seq) of schizont‐stage WT + PfH3p‐HA parasites. Downstream differential peak‐calling analysis was performed using MACS2 25: Comparisons included PfH3p‐HA signal relative to the ChIP input, PfH3p‐HA signal relative to the PfH3n, and PfH3n enrichment over ChIP input.

**Figure 4:**
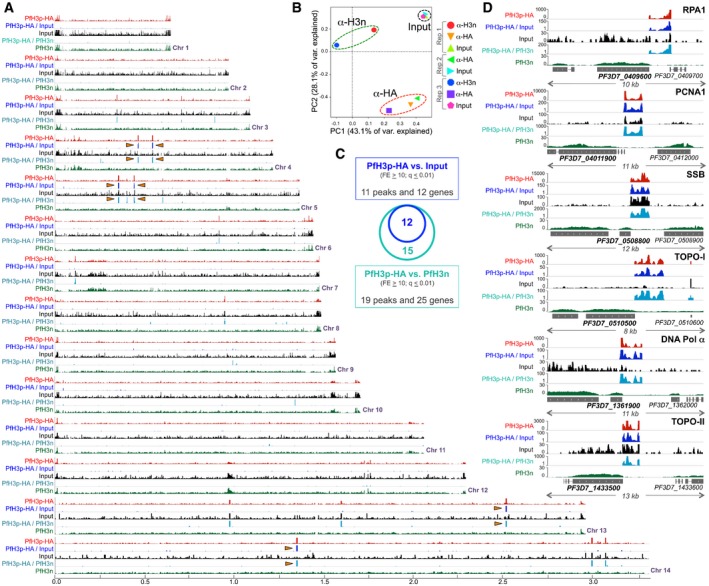
In *Plasmodium falciparum,* clipped histone H3 is targeted to the 5′UTR of genes regulating DNA replication Genome‐wide distribution of ectopically expressed PfH3p‐HA in *P. falciparum* schizont stages is represented as fold enrichment of PfH3p‐HA ChIP‐seq signal over input (PfH3p‐HA/input in blue; *y*‐axis scale 1–10) or over PfH3n (PfH3p‐HA/input in teal; *y*‐axis scale 1–10) calculated using MACS2. The coverage of PfH3p‐HA (red; *y*‐axis scale 0–50), input (black; *y*‐axis scale 0–30), and PfH3n (green; *y*‐axis scale 0–30) is also shown and represents average reads per million over 1,000 nt bins of the genome. The *x*‐axis represents chromosome size in Mb. Major MACS2‐derived peaks identified in all replicates are indicated using an orange arrowhead.Principal component analysis of the different ChIP‐seq replicates (bigwig files derived from deduplicated bam files) was performed using the plotPCA function of deepTools on a multibigwig summary file, over 150 nt bins. The Eigenvalues of the top two principal components PC1 and PC2 are shown, and meaningful clustering of replicates is highlighted. Rep = biological replicate.Summary of the PfH3p‐enriched peaks—and corresponding genes—identified in the PfH3p‐HA versus input or PfH3p‐HA versus PfH3n comparisons using MACS2 peak‐calling analysis. The overlap between the two comparisons is indicated using a Venn diagram. FE = fold enrichment; *q*‐value represents the Benjamini and Hochberg false discovery rate.The genomic context of the six peaks identified in all three PfH3p‐HA ChIP‐seq replicates is shown. The fold enrichment of PfH3p‐HA ChIP‐seq signal over input (PfH3p‐HA/input; blue) or PfH3n (PfH3p‐HA/PfH3n; teal) is shown, as are the coverage plots of PfH3p‐HA (red), PfH3n (green), and input (black), which are represented as average reads per million over 1,000 nt bins of the genome. DNA replication genes: replication protein A 1 (RPA1), proliferating cell nuclear antigen 1 (PCNA1), single‐stranded DNA‐binding protein (SSB), topoisomerase I (TOPO‐I), DNA polymerase alpha subunit (DNA pol a), and topoisomerase II (TOPO‐II) are highlighted and their directionality indicated. Note that the data in part D correspond to Biological Replicate 1.

First, we visualized the genome‐wide distribution of the anti‐HA ChIP‐seq signal (labeled PfH3p‐HA) and its fold enrichment (FE) relative to either the ChIP input (labeled PfH3p‐HA/input) or PfH3n (labeled PfH3p‐HA/PfH3n) using custom scripts and the IGV browser 26, and observed that fewer than 20 genomic regions were enriched for PfH3p‐HA across all 14 *P. falciparum* chromosomes (Fig 4A). The clustering of biological replicates for each ChIP‐seq condition in a principal component analysis (Fig 4B), and the Pearson correlation coefficients between replicates in ChIP‐seq as well as for MACS2‐derived FE profiles (Appendix Fig S3), shows a high degree of consistency between replicates. Next, using MACS2‐based peak‐calling analysis, we identified 11 peaks that overlapped between the PfH3p‐HA versus input comparison (Table EV3A) and the PfH3p‐HA versus PfH3n comparison (Dataset EV1A), at a cutoff of 10 FE and a *P*‐value of 0.01 after adjusting for the false discovery rate 27. These 11 peaks correspond to 12 genes that are listed in Table 1 along with the position of the peak relative to the coding sequence (cds). Of note, six of the 12 H3p‐enriched genes encode for proteins that regulate DNA replication and repair: replication protein A type 1 (RPA1), proliferating cell nuclear antigen 1 (PCNA1), single‐stranded DNA‐binding protein (SSB), topoisomerase I (TOPO‐I), topoisomerase II (TOPO‐II), and DNA polymerase alpha subunit (DNA pol α). Importantly, the H3p signal for these regions always begins within the 5′UTR and spans a region of approximately 1.5 kb, including up to 100 bp of the cds (Fig 4D and Appendix Fig S4, and Table 1). We also performed gene ontology (GO) analysis of the PfH3p‐HA enriched genes (Table EV3B and Dataset EV1A) and identified GO terms related to DNA replication, DNA binding, and DNA‐dependent functionality (e.g., regulation of DNA metabolic process, DNA topological change, DNA replication, DNA topoisomerase activity, DNA replisome, and replication fork) as being enriched.

**Table 1:** List of PfH3p‐HA peaks and their *Plasmodium falciparum* genomic location

Peak_name^a^	Location			Length (bp)	Gene_ID (Strand^b^)	Position (5′UTR, cds, 3′UTR)	Gene product
	Chr.	Start	End				
S.all.HAvI.2_peak_5	4	534,319	535,814	1,496	PF3D7_0411900 (−)	cds, 5′UTR	**DNA polymerase alphasubunit**
					PF3D7_0412000 (+)	5′UTR	LITAF‐like zinc finger protein, putative
S.all.HAvI.2_peak_7	5	364,306	365,998	1,693	PF3D7_0508800 (−)	cds, 5′UTR	**Single‐stranded DNA‐binding protein**
S.all.HAvI.2_peak_8	5	448,393	450,065	1,673	PF3D7_0510500 (−)	cds, 5′UTR	**Topoisomerase I**
S.all.HAvI.2_peak_15	14	133,7159	133,8757	1,599	PF3D7_1433500 (−)	cds, 5′UTR	**DNA topoisomerase 2**
S.all.HAvI.2_peak_14	13	248,3093	248,4802	1,710	PF3D7_1361900 (−)	cds, 5′UTR	**Proliferating cell nuclear antigen 1**
S.all.HAvI.2_peak_4^c^	4	456,175	457,042	868	PF3D7_0409700 (−)	cds, 5′UTR	**Replication protein A1, large subunit**
					PF3D7_0409600 (+)	5′UTR	Peptide chain release factor 2
S.all.HAvI.2_peak_9	7	514,911	515,153	243	PF3D7_0711700 (−)	cds	Erythrocyte membrane protein 1, PfEMP1
S.all.HAvI.2_peak_11	9	103,795	104,047	253	PF3D7_0902300 (−)	cds	Serine/threonine protein kinase, FIKK
S.all.HAvI.2_peak_13	13	973,880	974,032	153	PF3D7_1323400 (−)	5′UTR	60S ribosomal protein L23
S.all.HAvI.2_peak_1	2	275,544	275,782	239	PF3D7_0206900 (−)	cds	Merozoite surface protein 5
S.all.HAvI.2_peak_3^c^	4	455,551	456,010	460	PF3D7_0409600 (−)	cds, 5′UTR	**Replication protein A1, large subunit**
					PF3D7_0409700 (+)	5′UTR	Peptide chain release factor 2

Bold are indicated the genes that encoded for proteins implicated in DNA replication and repair.

a Peak_name was derived from Table EV3A.

b Indicates the strand corresponding to the coding sequence.

c S.all.HAvI.2_peak_4 and S.all.HAvI.2_peak_3 localize to the same genomic region.

To determine whether the observed PfH3p‐HA localization pattern was parasite stage‐specific, we performed ChIP‐seq analysis of ring‐stage WT + PfH3p‐HA parasites using anti‐HA and anti‐PfH3n antibodies and observed a strong correlation between the schizont and ring biological replicates for PfH3p‐HA (Appendix Fig S5). Furthermore, MACS2 peak‐calling analysis identified only six peaks that overlapped between the PfH3p‐HA versus input comparison (Dataset EV2) and the PfH3p‐HA versus PfH3n comparison (Dataset EV3), at a cutoff of 10 FE and *P*‐value of 0.01 after adjusting for false discovery rate. These corresponded to the six replication genes RPA1, PCNA1, SSB, TOPO‐I, TOPO‐II, and DNA pol α (Appendix Fig S6). Taken together, our ChIP‐seq data further support a *trans‐* model of histone H3p incorporation into nucleosomes and reveal the existence of a novel mechanism in *P. falciparum* that can recruit pre‐existing clipped histone H3 to specific genomic loci.

## Discussion

Here, we report a novel type of histone PTM in malaria parasites that involves a nuclear protease. We observe histone H3 clipping during asexual blood development of *P. falciparum* generating two closely related forms of truncated H3, PfH3int and PfH3p. Although we call PfH3int the intermediate form, it may represent the same truncated form as PfH3p but with distinct PTM status, which is supported by the detection of the doublet by anti‐H3K23ac antibodies but not by anti‐H3K27me3 antibodies (see Fig 1C). We also demonstrate that only a small number of genes have nucleosomes that contain clipped PfH3p‐HA. Strikingly, the majority of genes that show enriched PfH3p‐HA in their 5′UTR region are associated with DNA metabolism. We speculate that *P. falciparum* DNA replication genes are marked by this truncated form of histone H3 to provide a specific mechanism to coordinate DNA synthesis and subsequent karyokinesis during the process of parasite multiplication, i.e., schizogony. The observed association may therefore represent a malaria parasite‐specific adaptation that does not exist in other eukaryotic species. It will be interesting to see whether other closely related Apicomplexan parasites such as *Toxoplasma* spp. and *Babesia* spp. utilize a similar regulatory mechanism.

Histone H3 N‐terminal truncation at different sites has been reported to permanently remove epigenetic histone PTMs in several eukaryotes (for a review, see ref. 19, which in turn results in histone turnover or gene regulation). In these published cases, histone H3 clipping phenotypes were not linked to DNA replication blockage but affected, for example, differentiation of mouse embryonic stem cells 20 and the expression of stationary‐phase genes in *S. cerevisiae* 21. Furthermore, most of these studies did not even consider if the protease could operate in *trans‐*, the default assumption was that it acted in *cis‐*. In contrast, our experimental data from malaria parasites support a cellular mechanism that incorporates ectopically clipped histone H3 into chromatin regions at particular genome loci: *P. falciparum* chaperones specific to clipped histone H3 may target PfH3p‐containing pre‐assembled nucleosomes to specific gene loci or chromatin remodeling enzymes such as the SWI/SNF complex, and others 28 may exchange full‐length histone H3 for PfH3p at replication gene loci. Indeed, in the absence of a selection process for clipped histones, we would have expected the ectopically expressed PfH3p‐HA to be distributed throughout the *P. falciparum* genome. Nevertheless, we cannot rule out that histone H3 clipping occurs in the chromatin context: This would require the targeting of the histone H3 protease to specific genomic regions, which can be addressed upon protease identification.

Thus far, diverse families of proteases have been implied in histone H3 clipping, including cathepsin L‐type cysteine proteases 20, tryptase 22, glutamate dehydrogenase 29, Jumonji C domain (JmjC)‐containing proteins, JMJD5 and JMJD7 30, and an unidentified yeast enzyme that has intrinsic aminopeptidase and endopeptidase activity 21. Interestingly, Duncan *et al* 20 reported that covalent histone modifications modulate cathepsin L activity. Our data also point to a histone modification upstream of the truncated region at lysine 27 that may contribute to H3 N‐terminal clipping. The cleaved H3 shows H3K27me3, a methylation mark that was previously not reported for malaria parasites. In *P. falciparum,* the utilization of a variety of protease inhibitors in the *in vitro* proteolysis assay hinted at the presence of a cathepsin C‐type exo‐cysteine peptidase in parasite nuclear extracts that cleaves histone H3 at its N‐terminus. Interestingly, in T lymphocytes and natural killer cells, cathepsin C is a lysosomal protease that has been implicated in processing pro‐granzymes into proteolytically active forms 31. It also plays a role in epithelial differentiation 32, with genetic mutations of the cathepsin C gene CTSC causing the autosomal recessive disorder Papillon–Lefèvre syndrome 33.

The *P. falciparum* genome (www.plasmodb.org/plasmo) encodes three cysteine proteases of the cathepsin C subtype, also known as dipeptidyl aminopeptidase (DPAP). Two of them, PfDPAP1 (*PF3D7_1116700*) and PfDPAP3 *(PF3D7_0404700),* are expressed in the cytoplasm of asexual blood stages and are involved in vacuolar protein degradation and erythrocyte invasion, respectively 34, 35, while PfDPAP2 (*PF3D7_1247800*) expression was observed in sexual‐stage parasites 36. Furthermore, a cathepsin L‐like protease has not been annotated in the PlasmoDB genome database (https://plasmodb.org). Biochemical fractionation of nuclear extracts followed by mass spectrometry may reveal the nature of the candidate clipping protease, which is beyond the scope of this work.

In conclusion, the discovery of a novel type of PTM in malaria parasites predicts the existence of an added layer of epigenetic regulation in this highly complex pathogen. Given that the N‐terminal region of histone H3 (amino acids 1–21) is a key regulator of gene expression in *P. falciparum*, especially of virulence genes, regulating the N‐terminal tail by proteolysis may trigger a so‐far unknown response, possibly cell cycle progression or overcoming a mitotic checkpoint to enable karyokinesis. Furthermore, it opens up novel avenues for reducing malaria mortality. If histone clipping is conserved during *P*. *falciparum* proliferative stages in the mosquito and human liver, specific inhibitors of the clipping process may target pre‐ and intra‐erythrocytic development as well as mosquito stages. Finally, the identification of the gene that codes for the nuclear clipping protease is of particular interest to investigate the biological role of histone clipping during the various developmental stages of this major human pathogen.

## Materials and Methods

### Parasite culture

*Plasmodium falciparum* parasites of the laboratory‐adapted 3D7 strain were cultured following standard protocols 37. Parasites were synchronized with 5% sorbitol and plasmion on a regular basis 38. Transfection of ring‐stage parasites of the 3D7 genotype using 100 μg of the pARL‐H3K22‐HA(3X) vector was performed as described previously 39. Parasites carrying the pARL‐H3K22‐HA(3X) plasmid (described below) were selected with 10 nM WR99210 (Walter Reed Army Institute of Research).

### Generation of transgenic parasites

Transgenic parasites expressing a truncated version of *P. falciparum* histone H3 (amino acids S22–S136) tagged with three haemagglutinin A [HA(3X)] epitopes at its C‐terminus were generated as follows. First, the vector pARL‐SSG (in which the ORF of a Stevor gene is fused to green fluorescent protein (GFP) and expressed from the CRT promoter 40) was digested with the *XhoI* and *KpnI* restriction enzymes to release the *Stevor* and GFP cds from the pARL vector backbone. Next, the H3S22‐S136‐HA(3X) cassette was amplified by PCR using genomic DNA from the wild‐type 3D7 strain as template and the following sense and antisense primers:

*XhoI*‐H3K21 (5′‐CCGCTCGAGTCAAAGCAGCAAGGAAATCA‐3′) and *KpnI*‐HA(3X) (5′‐GGGGTACCTTATGCATAATCTGGTACATCATATGGATATGCATAATCTGGT ACATCATATGGATATGCATAATCTGGTACATCATATGGATAAGATCTTTCCCACGAATACG‐3′) to amplify the truncated version of histone H3. This insert was digested with *XhoI* and *KpnI* enzymes and ligated to the pARL vector backbone to obtain the transfection vector pARL‐H3S22‐HA(3X), which was amplified for transfection into 3D7. Drug‐resistant parasites emerged after 4 weeks of continuous culture in the presence of 5 nM WR92210.

### Nuclear and cytoplasmic extract preparation

Nuclear and cytoplasmic extracts from synchronized asexual stages (ring, trophozoites, and schizonts) were prepared as described previously 41. In order to prepare nuclear extracts for use in the *in vitro* proteolysis assays, buffers were *not* supplemented with protease inhibitors.

### Mononucleosome preparation

Mononucleosome preparation was carried out as described previously 41. Briefly, schizont‐stage parasites of WT or WT + PfH3p‐HA were isolated from infected erythrocytes by saponin lysis, resuspended in 1 ml of buffer A (10 mM HEPES, pH 7.9, 10 mM KCl, 1.5 mM MgCl_2_) supplemented with protease inhibitors (Complete, Roche), and incubated for 30 min at 4°C. Parasites were lysed with 200 strokes in a prechilled Dounce homogenizer and nuclei collected by centrifugation, cleaned through a 0.34 M sucrose cushion, and resuspended in 1 ml of prechilled buffer A supplemented with 2 mM CaCl_2_ plus protease inhibitors. Mononucleosomes were prepared from freshly isolated nuclei by digestion with 4 U MNase (NEB) for different amounts of time at 33°C; digestion was stopped by adding 10 mM EGTA pH 8.0. After spinning out debris at 18,800 *g* for 15 min at 4°C, the supernatant was analyzed for the presence of mononucleosomes by extracting DNA and resolving it on a 2% agarose gel stained with ethidium bromide. Typically, after 30 min of MNase digestion, the population contained 95% mononucleosomes.

### Antibodies and immunoblotting

The following commercial antibodies (Ab) were used for immunoblotting assays: Anti‐histone H3 C‐terminal Ab (Abcam Ab1791) were used at a 1:150,000 dilution, anti‐histone H3 N‐terminal Ab (Sigma‐9289) were used at 1:1,000 dilution, anti‐histone H4 Ab (Santa Cruz sc‐8658‐R) were used at 1:3,000 dilution, anti‐H3K9me3 Ab (Millipore 07‐442) were used at a 1:3,000 dilution, anti‐H3K18ac Ab (Millipore 07‐354) were used at a 1:40,000 dilution, anti‐H3K23ac Ab (Abcam ab46982) were used at a 1:100,000 dilution, anti‐H3K27me3 Ab (Abcam ab6147) were used at a 1:2,000, anti‐H3K27ac Ab (Millipore 07‐360) were used at a 1:40,000 dilution, anti‐GST Ab (Invitrogen 13‐6700) were used at a 1:50,000 dilution, and anti‐HA Ab (Roche 12CA5) were used at a 1:2,000 dilution. After incubation with horseradish peroxidase‐conjugated secondary antibodies, membranes were developed with SuperSignal West Pico Chemiluminescent Substrate (Pierce) according to the manufacturer's instructions.

### Histone extraction and mapping of H3 cleavage site

Acid extraction of histones from synchronized asexual stages (ring, trophozoites, and schizonts) of *P. falciparum* was carried out as previously described 41. In brief, infected erythrocytes were harvested by centrifugation at 500 *g* for 5 min and parasites isolated by saponin lysis. The resulting parasite pellet was resuspended in 1 ml of lysis buffer (10 mM Tris pH 8, 15 mM KCl, 1.5 mM MgCl_2_, 1 mM DTT, 0.25% NP‐40) supplemented with protease inhibitors (Complete, Roche) and incubated for 30 min at 4°C. Parasites were then lysed with 200 strokes in a prechilled Dounce homogenizer, and the nuclei were collected by centrifugation at 9,600 *g* for 15 min and washed once with 10 volumes of 0.3 M NaCl and twice with 10 volumes of 0.5 M NaCl. Histones were extracted by incubating the samples with 10 volumes of 0.25 M HCl overnight at 4°C. The extracts were centrifuged at 18,800 *g* for 30 min to remove insoluble debris and histones precipitated with eight volumes of cold acetone (incubation overnight at −20°C) and centrifugation at 13,800 *g* for 30 min at 4°C. The final pellets were air‐dried, resuspended in 50 mM Tris–HCl pH 8.8, and stored at −80°C.

To determine the cleavage site of PfH3, equal amounts of acid‐purified *P. falciparum* histones were separated by 18% SDS–PAGE and transferred onto nitrocellulose membranes. Membranes were probed with commercially available antibodies against core histone H4, core histone H3 (C‐terminal), H3K9me3, H3K18ac, H3K23ac, H3K27me3, and H3K27ac. Alternatively, histone H3 and its processed forms were excised from the nitrocellulose membrane, after probing with core histone H3 antibodies, and subjected to Edman degradation as described previously 42.

### Mass spectrometry

In‐gel digestion of proteins was carried out according to the manufacturer's manual (Pierce, Rockford, IL). Briefly, the proteins of interest were separated on a denaturing polyacrylamide gel and stained with Coomassie blue. The band of interest was excised, destained, and disulfide bonds reduced with Tris 2‐carboxyethylphosphine (TCEP) and alkylated with iodoacetamide. The gel pieces were then dehydrated with acetonitrile and rehydrated with 100 ng trypsin (Promega, Madison, WI) in 25 mM ammonium bicarbonate solution and incubated at 37°C overnight. The tryptic fragments were extracted from the gel by adding 1% trifluoroacetic acid. Next, the peptides were purified with a C18 reversed‐phase mini‐column filled into a micropipette tip, i.e., ZipTip C18 (Millipore, Bedford, MA). Purified peptides were co‐crystallized with α‐cyano‐4‐hydroxycinnamic acid matrix (Applied Biosystems, Foster City, CA, USA) on a matrix‐assisted laser desorption ionization (MALDI) target plate. Both mass spectrometry (MS) and MS/MS spectra were acquired in a MALDI–time‐of‐flight (MALDI–TOF/TOF) mass spectrometer (Applied Biosystems 4800 Proteomics Analyzer) in the reflector mode. Calibration was updated before each acquisition using a standard peptide mixture according to the instrument's protocol. Protein identification was performed using the GPS Explorer software (Applied Biosystems) with the MASCOT search engine (Matrix Science). The search was performed against the NCBI‐nr 20080502 database (6493741 sequences; 2216039219 residues), and the Mascot significance threshold was set at *P* = 0.05. Individual ions scores > 33 indicated identity or extensive homology and were considered to be significant.

### Tandem Mass Spectrometry Analysis (LC/ESI‐MS/MS) to identify post‐translational modifications of histones

Protein bands were carefully excised from a Coomassie‐stained SDS–polyacrylamide gel (SDS–PAG) and destained with 50% (v/v) methanol, 5% (v/v) acetic acid for 12 h. The destained gels were washed with deionized water, soaked for 10 min in 100 mM ammonium bicarbonate, cut into small pieces, dehydrated with 100% acetonitrile, and vacuum‐dried. Proteins were reduced with 10 mM DTT and S‐alkylated cysteine with 100 mM iodoacetamide in 100 mM ammonium bicarbonate. In‐gel digestion was performed by adding 30 μl of modified porcine trypsin solution (20 ng/μl) in 50 mM ammonium bicarbonate followed by overnight incubation at room temperature. Peptides were extracted with 50% (v/v) acetonitrile, 5% (v/v) formic acid twice for 30 min each with sonication. The extracts were dried under vacuum and resuspended in 20 μl of 0.1% formic acid.

HPLC/MS/MS analysis of peptides was carried out using an integrated nano‐LC‐ESI_MS/MS system: It consists of a nanoACQUITY ultraperformance liquid chromatography (UPLC; Waters Corporation) coupled to a Q‐ToF Synapt G2 High Definition Mass Spectrometer (Waters Corporation) equipped with a NanoLockSpray ion source. The mass spectrometer was calibrated with a NaCsI solution (mass range: 50–2,000 Da) and operated in ESI positive V‐mode at a resolution of 10,000 fwhh (full with at half height). Spectra were acquired in automated mode using data‐dependent acquisition (DDA). [Glu^1^]‐fibrinopeptide B solution (100 fmol/μl) was infused through the reference sprayer of the NanoLockSpray source at a flow rate of 0.5 μl/min and was sampled at 30‐s intervals during the acquisition. MS survey scans of 1 s over the *m*/*z* range 300–1,600 were used for peptide detection followed by two MS/MS scans of 2 s each (*m/z* 50–2,000) of detected precursors. Collision energies were automatically adjusted based upon the ion charge state and the mass. The five most intense precursor ions of charge 2^+^, 3^+^, or 4^+^ were interrogated per MS/MS switching event. Dynamic exclusion for 60 s was used to minimize multiple MS/MS events for the same precursor. The data were post‐acquisition lock mass corrected using the doubly protonated monoisotopic ion of [Glu^1^]‐fibrinopeptide.

Data‐dependent acquisition raw data files were processed and searched using the ProteinLynx Global Server, version 2.4, software (PLGS; Waters Corporation). The default parameter settings included: perform lock spray calibration with a lock mass tolerance of 0.1 Da, background substract type of adaptive, and deisotoping type of medium. PLGS was configured to automatically output PKL files which were subsequently database searched by MASCOT search algorithm (version 1.6b9, Matrix Science, London, UK) available at http://www.matrixscience.com. Mascot searches were conducted using the *P. falciparum* subset (Feb 2011, 16,710 sequences) of the National Center for Biotechnology Information non‐redundant database (NCBI‐nr, http://www.ncbi.nih.gov). Trypsin was set as the digest reagent, and one missed cleavage site was allowed. Mass tolerances of 30 ppm and 0.6 Da were used for precursor and product ions, respectively. Fixed modifications: carbamidomethyl‐cysteine. Variable modifications: oxidation (M), deamidation (Q, N), acetylation (K, N‐terminal), methylation, and dimethylation (K). Peptide matches with Mascot scores exceeding the 95% level of confidence were accepted as correct matches. Ion score is −10*Log(*P*) where *P* is the probability that the observed match is a random event. The threshold score was 40 for *P* < 0.05.

### Purification of recombinant PfH3 and PfH2A histones

The PfH3 ORF (*PF3D7_0610400*; START to STOP codon = 408 bp) was PCR‐amplified using the following primers: PfH3‐*EcoRI* (5′‐GGAATTCCATGGCAAGAACTAAACAAACA‐3′) and PfH3‐*XhoI* (5′‐CCGCTCGAGTGATCTTTCTCCACGGATACG‐3′) and inserted into the pGEX‐T1 vector (GE Healthcare), downstream of the glutathione S‐transferase (GST) sequence. Similarly, the ORF of PfH2A (*PF3D7_0617800*; START to STOP codon = 396 bp) was PCR‐amplified using the following primers: PfH2A‐*EcoRI* (5′‐GGAATTCCATGTCAGCAAAAGGAAAAACT‐3′) and PfH2A‐*XhoI* (5′‐CCGCTCGAGTTAATAATCTTGATTGGCAGT‐3′) and inserted into pGEX4‐T1, downstream of the GST‐coding sequence. The resulting constructs were transformed into *Escherichia coli* DH5α cells and grown at 30°C in Luria Bertani (LB) medium with 100 μg/ml ampicillin, under good aeration to an OD of 0.4–0.6, at which point GST‐tagged protein expression was induced with 2 mM IPTG for 2 h at 30°C. Induced cells were harvested by centrifugation at 2,400 *g* for 10 min. The cell pellets were resuspended in PBS, disrupted by sonication on ice and centrifuged for 20 min at 6,900 *g* to get rid of unbroken cells and other debris. GST‐tagged proteins were purified using Glutathione Sepharose 4B beads (GE Healthcare) according to the manufacturer's instructions and eluted in 300 μl buffer. Protein concentration was determined using a Bradford assay (*24*). 1–2 μg of GST‐PfH3 or GST‐PfH2A was resolved using 12% SDS–PAGE, and protein purity was determined by Coomassie blue staining.

### *In vitro* proteolysis assays

*In vitro* proteolysis assays were performed as described previously 20. Briefly, 45 μg of *P. falciparum* nuclear extract (as a source of proteases) was mixed with 1.5 μg of recombinant protein (GST‐PfH3 or GST‐PfH2A) in a 60 μl reaction volume in a buffer containing 10 mM HEPES, 10 mM KCl, 1.5 mM MgCl_2_, 0.34 M sucrose, 10% glycerol, and 5 mM β‐mercaptoethanol and incubated for 3 h at 37°C. The resulting products were resolved on a 12% SDS–PAGE gel and the proteolytic pattern analyzed by immunoblotting with HRP‐conjugated anti‐GST antibodies. For Fig 2D, inhibitors specific for different families of proteases were used at a concentration of 1 mM AEBSF, 100 μM antipain, 220 μM bestatin, 100 μM chymostatin, 100 μM E‐64, or 10 mM EDTA.

### Immunofluorescence microscopy

Indirect immunofluorescence assays were performed as previously described 43 with anti‐HA (1:2,000 dilution) or anti‐histone H3 C‐terminal (1:2,000 dilution) antibodies. DAPI was used to stain DNA, and images were acquired using a Olympus Fluoview FV300 microscope, software v4.3.

### Co‐immunoprecipitation assays

To improve solubility for co‐immunoprecipitation assays, the mononucleosome preparation from WT or WT + PfH3p‐HA parasites was supplemented with 0.05% Nonidet P‐40 (NP‐40) and 200 mM NaCl followed by an additional extraction using RIPA buffer (Sigma R0278). Prior to immunoprecipitation, mononucleosomes were precleared by incubation with protein‐G agarose beads for 1–3 h at 4°C. For immunoprecipitation, 200 μl of precleared mononucleosomes was incubated with 0.5 μg anti‐HA antibodies (Roche) overnight at 4°C with rotation. Subsequently, 50 μl of protein‐G agarose beads was added to each immunoprecipitation reaction and incubated for 3 h at 4°C with rotation. Bound material was washed thrice for 5 min with equilibration buffer (50 mM Tris pH 8.0, 150 mM NaCl, 5 mM EDTA pH 8.0, 0.05% NP‐40) supplemented with protease inhibitors and eluted by boiling for 5 min in SDS–PAGE Laemmli buffer (37.9 mM Tris pH 6.8, 13.15% glycerol, 0.05% SDS, 0.005% bromophenol blue, and 355 mM β‐mercaptoethanol). Immunoprecipitated proteins were analyzed using immunoblotting with anti‐HA and anti‐histone H4 antibodies.

### Chromatin immunoprecipitation and next‐generation sequencing

Chromatin immunoprecipitation (ChIP) assays were performed as described previously 44 with anti‐HA (Roche, 12CA5), anti‐histone H3 N‐terminal (H9298 Sigma), or mouse IgG (Sigma I5381) antibodies. Immunoprecipitated DNA and DNA corresponding to the ChIP input were using the Illumina Library Preparation Kit (Diagenode) according to the manufacturer's instructions. 4–12 libraries were multiplexed and run on an Illumina HiSeq 2500 or a NextSeq 500 as a single‐end run of 100 nt. The resulting fastq files were analyzed as described in the “Analysis of NGS data” section. A minimum of two biological replicates was evaluated for each ChIP assay.

### Analysis of NGS data

The analysis of ChIP‐seq datasets was performed using several command line tools: BWA‐MEM 45, SAMtools 46, deepTools v2.4.0, bedtools v2.26.0 47, and MACS2 25. First, quality control of fastq files was performed using the FastQC software (https://www.bioinformatics.babraham.ac.uk/projects/fastqc/). Next, sequencing reads were mapped to the *P. falciparum* 3D7 genome (v3, GeneDB) using BWA‐MEM under default settings to generate SAM files, which were further processed to the bam format using SAMtools. Before running MACS, the correlation between biological replicates was determined using the Pearson correlation and principal component analysis functionalities of deepTools and the normalized coverage of reads for each bam file calculated using bedtools. Thereafter, quality‐filtered bam files (Q10 or Q20) served as the starting point for differential peak‐calling analysis using the call peak function in MACS2. Fold enrichment of ChIP signal of treatment relative to the control was determined using the bdgcmp function in MACS2. The resulting bedgraphs were converted to bigWigs using bedGraphToBigWig 48 and the correlation between bigwig files analyzed using deepTools. Note that the mapping properties of the fastq and bam files generated in this study and related statistics are provided in Dataset EV4.

## Author contributions

AS and RH‐R conceived and designed the experiments; AMH‐S performed mass spectrometry experiments, immunoblot, and ChIP experiments; MV developed the *in vitro* proteolysis assays; SSV and RMM performed the ChIP‐seq experiments; SSV and CRM analyzed the ChIP‐seq data; DL‐A performed immunofluorescence assays; BX‐C, GRM, and PC obtained the recombinant proteins; and SSV, AS, and RH‐R wrote the manuscript. All authors read and approved the final manuscript.

## Conflict of interest

The authors declare that they have no conflict of interest.

## Supporting information



Appendix

Table EV1

Table EV2

Table EV3

Dataset EV1

Dataset EV2

Dataset EV3

Dataset EV4

Source Data for Appendix

Review Process File

Source Data for Figure 1

Source Data for Figure 2

Source Data for Figure 3

## Data Availability

The fastq files supporting the results of this article are available in the EMBL‐EBI European Nucleotide Archive (ENA: PRJEB18114; Sample group: ERG011046): http://www.ebi.ac.uk/ena/data/view/PRJEB18114.

## References

[1] WHO 2015 Fact sheet: world malaria report 2015. http://www.who.int/malaria/publications/world-malaria-report-2015/report/en/

[2] Scherf A , Lopez‐Rubio JJ , Riviere L (2008) Antigenic variation in *Plasmodium falciparum* . Annu Rev Microbiol 62: 445–470 18785843 10.1146/annurev.micro.61.080706.093134

[3] Cui L , Miao J (2010) Chromatin‐mediated epigenetic regulation in the malaria parasite *Plasmodium falciparum* . Eukaryot Cell 9: 1138–1149 20453074 10.1128/EC.00036-10 PMC2918932

[4] Freitas‐Junior LH , Hernandez‐Rivas R , Ralph SA , Montiel‐Condado D , Ruvalcaba‐Salazar OK , Rojas‐Meza AP , Mancio‐Silva L , Leal‐Silvestre RJ , Gontijo AM , Shorte S *et al* (2005) Telomeric heterochromatin propagation and histone acetylation control mutually exclusive expression of antigenic variation genes in malaria parasites. Cell 121: 25–36 15820676 10.1016/j.cell.2005.01.037

[5] Duraisingh MT , Voss TS , Marty AJ , Duffy MF , Good RT , Thompson JK , Freitas‐Junior LH , Scherf A , Crabb BS , Cowman AF (2005) Heterochromatin silencing and locus repositioning linked to regulation of virulence genes in *Plasmodium falciparum* . Cell 121: 13–24 15820675 10.1016/j.cell.2005.01.036

[6] Josling GA , Llinas M (2015) Sexual development in *Plasmodium* parasites: knowing when it's time to commit. Nat Rev Microbiol 13: 573–587 26272409 10.1038/nrmicro3519

[7] Lopez‐Rubio JJ , Mancio‐Silva L , Scherf A (2009) Genome‐wide analysis of heterochromatin associates clonally variant gene regulation with perinuclear repressive centers in malaria parasites. Cell Host Microbe 5: 179–190 19218088 10.1016/j.chom.2008.12.012

[8] Malmquist NA , Moss TA , Mecheri S , Scherf A , Fuchter MJ (2012) Small‐molecule histone methyltransferase inhibitors display rapid antimalarial activity against all blood stage forms in *Plasmodium falciparum* . Proc Natl Acad Sci USA 109: 16708–16713 23011794 10.1073/pnas.1205414109 PMC3478629

[9] Malmquist NA , Sundriyal S , Caron J , Chen P , Witkowski B , Menard D , Suwanarusk R , Renia L , Nosten F , Jiménez‐Díaz MB *et al* (2015) Histone methyltransferase inhibitors are orally bioavailable, fast‐acting molecules with activity against different species causing malaria in humans. Antimicrob Agents Chemother 59: 950–959 25421480 10.1128/AAC.04419-14 PMC4335868

[10] Trenholme K , Marek L , Duffy S , Pradel G , Fisher G , Hansen FK , Skinner‐Adams TS , Butterworth A , Ngwa CJ , Moecking J *et al* (2014) Lysine acetylation in sexual stage malaria parasites is a target for antimalarial small molecules. Antimicrob Agents Chemother 58: 3666–3678 24733477 10.1128/AAC.02721-13 PMC4068603

[11] Miao J , Fan Q , Cui L , Li J (2006) The malaria parasite *Plasmodium falciparum* histones: organization, expression, and acetylation. Gene 369: 53–65 16410041 10.1016/j.gene.2005.10.022

[12] Trelle MB , Salcedo‐Amaya AM , Cohen AM , Stunnenberg HG , Jensen ON (2009) Global histone analysis by mass spectrometry reveals a high content of acetylated lysine residues in the malaria parasite *Plasmodium falciparum* . J Proteome Res 8: 3439–3450 19351122 10.1021/pr9000898

[13] Dastidar EG , Dzeyk K , Krijgsveld J , Malmquist NA , Doerig C , Scherf A , Lopez‐Rubio JJ (2013) Comprehensive histone phosphorylation analysis and identification of pf14‐3‐3 protein as a histone h3 phosphorylation reader in malaria parasites. PLoS One 8: e53179 23308157 10.1371/journal.pone.0053179 PMC3538786

[14] Sindikubwabo F , Ding S , Hussain T , Ortet P , Barakat M , Baumgarten S , Cannella D , Palencia A , Bougdour A , Belmudes L *et al* (2017) Modifications at K31 on the lateral surface of histone H4 contribute to genome structure and expression in apicomplexan parasites. Elife 6: e29391 29101771 10.7554/eLife.29391 PMC5685513

[15] Lopez‐Rubio JJ , Gontijo AM , Nunes MC , Issar N , Hernandez Rivas R , Scherf A (2007) 5′ flanking region of var genes nucleate histone modification patterns linked to phenotypic inheritance of virulence traits in malaria parasites. Mol Microbiol 66: 1296–1305 18028313 10.1111/j.1365-2958.2007.06009.x PMC2228885

[16] Brancucci NM , Bertschi NL , Zhu L , Niederwieser I , Chin WH , Wampfler R , Freymond C , Rottmann M , Felger I , Bozdech Z *et al* (2014) Heterochromatin protein 1 secures survival and transmission of malaria parasites. Cell Host Microbe 16: 165–176 25121746 10.1016/j.chom.2014.07.004

[17] Kafsack BF , Rovira‐Graells N , Clark TG , Bancells C , Crowley VM , Campino SG , Williams AE , Drought LG , Kwiatkowski DP , Baker DA *et al* (2014) A transcriptional switch underlies commitment to sexual development in malaria parasites. Nature 507: 248–252 24572369 10.1038/nature12920 PMC4040541

[18] Azad GK , Swagatika S , Kumawat M , Kumawat R , Tomar RS (2018) Modifying chromatin by histone tail clipping. J Mol Biol 430: 3051–3067 30009770 10.1016/j.jmb.2018.07.013

[19] Dhaenens M , Glibert P , Meert P , Vossaert L , Deforce D (2015) Histone proteolysis: a proposal for categorization into ‘clipping’ and ‘degradation’. BioEssays 37: 70–79 25350939 10.1002/bies.201400118 PMC4305269

[20] Duncan EM , Muratore‐Schroeder TL , Cook RG , Garcia BA , Shabanowitz J , Hunt DF , Allis CD (2008) Cathepsin L proteolytically processes histone H3 during mouse embryonic stem cell differentiation. Cell 135: 284–294 18957203 10.1016/j.cell.2008.09.055 PMC2579750

[21] Santos‐Rosa H , Kirmizis A , Nelson C , Bartke T , Saksouk N , Cote J , Kouzarides T (2009) Histone H3 tail clipping regulates gene expression. Nat Struct Mol Biol 16: 17–22 19079264 10.1038/nsmb.1534 PMC3350865

[22] Melo FR , Wallerman O , Paivandy A , Calounova G , Gustafson AM , Sabari BR , Zabucchi G , Allis CD , Pejler G (2017) Tryptase‐catalyzed core histone truncation: a novel epigenetic regulatory mechanism in mast cells. J Allergy Clin Immunol 140: 474–485 28108335 10.1016/j.jaci.2016.11.044

[23] Perez‐Toledo K , Rojas‐Meza AP , Mancio‐Silva L , Hernandez‐Cuevas NA , Delgadillo DM , Vargas M , Martinez‐Calvillo S , Scherf A , Hernandez‐Rivas R (2009) *Plasmodium falciparum* heterochromatin protein 1 binds to tri‐methylated histone 3 lysine 9 and is linked to mutually exclusive expression of var genes. Nucleic Acids Res 37: 2596–2606 19270070 10.1093/nar/gkp115 PMC2677873

[24] Flueck C , Bartfai R , Volz J , Niederwieser I , Salcedo‐Amaya AM , Alako BT , Ehlgen F , Ralph SA , Cowman AF , Bozdech Z *et al* (2009) *Plasmodium falciparum* heterochromatin protein 1 marks genomic loci linked to phenotypic variation of exported virulence factors. PLoS Pathog 5: e1000569 19730695 10.1371/journal.ppat.1000569 PMC2731224

[25] Zhang Y , Liu T , Meyer CA , Eeckhoute J , Johnson DS , Bernstein BE , Nusbaum C , Myers RM , Brown M , Li W *et al* (2008) Model‐based analysis of ChIP‐Seq (MACS). Genome Biol 9: R137 18798982 10.1186/gb-2008-9-9-r137 PMC2592715

[26] Robinson JT , Thorvaldsdóttir H , Winckler W , Guttman M , Lander ES , Getz G , Mesirov JP (2011) Integrative genomics viewer. Nat Biotechnol 29: 24–26 21221095 10.1038/nbt.1754 PMC3346182

[27] Benjamini Y , Hochberg Y (1995) Controlling the false discovery rate: a practical and powerful approach to multiple testing. J R Stat Soc Series B Stat Methodol 57: 287–300

[28] Venkatesh S , Workman JL (2015) Histone exchange, chromatin structure and the regulation of transcription. Nat Rev Mol Cell Biol 16: 178–189 25650798 10.1038/nrm3941

[29] Mandal P , Chauhan S , Tomar RS (2014) H3 clipping activity of glutamate dehydrogenase is regulated by stefin B and chromatin structure. FEBS J 281: 5292–5308 25263734 10.1111/febs.13069

[30] Liu H , Wang C , Lee S , Deng Y , Wither M , Oh S , Ning F , Dege C , Zhang Q , Liu X *et al* (2017) Clipping of arginine‐methylated histone tails by JMJD5 and JMJD7. Proc Natl Acad Sci USA 114: E7717–E7726 28847961 10.1073/pnas.1706831114 PMC5604019

[31] Perišić Nanut M , Sabotič J , Jewett A , Kos J (2014) Cysteine cathepsins as regulators of the cytotoxicity of NK and T cells. Front Immunol 5: 616 25520721 10.3389/fimmu.2014.00616 PMC4251435

[32] Toomes C , James J , Wood AJ , Wu CL , Mccormick D , Lench N , Hewitt C , Moynihan L , Roberts E , Woods CG *et al* (1999) Loss‐of‐function mutations in the cathepsin C gene result in periodontal disease and palmoplantar keratosis. Nat Genet 23: 421–424 10581027 10.1038/70525

[33] Nagy N , Vályi P , Csoma Z , Sulák A , Tripolszki K , Farkas K , Paschali E , Papp F , Tóth L , Fábos B *et al* (2014) CTSC and Papillon‐Lefèvre syndrome: detection of recurrent mutations in Hungarian patients, a review of published variants and database update. Mol Genet Genomic Med 2: 217–228 24936511 10.1002/mgg3.61 PMC4049362

[34] Arastu‐Kapur S , Ponder EL , Fonović UP , Yeoh S , Yuan F , Fonović M , Grainger M , Phillips CI , Powers JC , Bogyo M (2008) Identification of proteases that regulate erythrocyte rupture by the malaria parasite *Plasmodium falciparum* . Nat Chem Biol 4: 203–213 18246061 10.1038/nchembio.70

[35] Deu E , Leyva MJ , Albrow VE , Rice MJ , Ellman JA , Bogyo M (2010) Functional studies of *Plasmodium falciparum* dipeptidyl aminopeptidase I using small molecule inhibitors and active site probes. Chem Biol 17: 808–819 20797610 10.1016/j.chembiol.2010.06.007 PMC2929396

[36] Tanaka TQ , Deu E , Molina‐Cruz A , Ashburne MJ , Ali O , Suri A , Kortagere S , Bogyo M , Williamson KC (2013) Plasmodium dipeptidyl aminopeptidases as malaria transmission‐blocking drug targets. Antimicrob Agents Chemother 57: 4645–4652 23836185 10.1128/AAC.02495-12 PMC3811399

[37] Trager W , Jensen JB (1976) Human malaria parasites in continuous culture. Science 193: 673–675 781840 10.1126/science.781840

[38] Lambros C , Vanderberg JP (1979) Synchronization of *Plasmodium falciparum* erythrocytic stages in culture. J Parasitol 65: 418–420 383936

[39] Crabb BS , Cowman AF (1996) Characterization of promoters and stable transfection by homologous and nonhomologous recombination in *Plasmodium falciparum* . Proc Natl Acad Sci USA 93: 7289–7294 8692985 10.1073/pnas.93.14.7289 PMC38976

[40] Przyborski JM , Miller SK , Pfahler JM , Henrich PP , Rohrbach P , Crabb BS , Lanzer M (2005) Trafficking of STEVOR to the Maurer's clefts in *Plasmodium falciparum*‐infected erythrocytes. EMBO J 24: 2306–2317 15961998 10.1038/sj.emboj.7600720 PMC1173160

[41] Cary C , Lamont D , Dalton JP , Doerig C (1994) *Plasmodium falciparum* chromatin: nucleosomal organisation and histone‐ like proteins. Parasitol Res 80: 255–258 8036241 10.1007/BF00932684

[42] Strahl BD , Ohba R , Cook RG , Allis CD (1999) Methylation of histone H3 at lysine 4 is highly conserved and correlates with transcriptionally active nuclei in Tetrahymena. Proc Natl Acad Sci USA 96: 14967–14972 10611321 10.1073/pnas.96.26.14967 PMC24756

[43] Mancio‐Silva L , Rojas‐Meza AP , Vargas M , Scherf A , Hernandez‐Rivas R (2008) Differential association of Orc1 and Sir2 proteins to telomeric domains in *Plasmodium falciparum* . J Cell Sci 121: 2046–2053 18525026 10.1242/jcs.026427

[44] Lopez‐Rubio JJ , Siegel TN , Scherf A (2013) Genome‐wide chromatin immunoprecipitation‐sequencing in *Plasmodium* . Methods Mol Biol 923: 321–333 22990789 10.1007/978-1-62703-026-7_23 PMC7116682

[45] Li H , Durbin R (2010) Fast and accurate long‐read alignment with Burrows‐Wheeler transform. Bioinformatics 26: 589–595 20080505 10.1093/bioinformatics/btp698 PMC2828108

[46] Li H , Handsaker B , Wysoker A , Fennell T , Ruan J , Homer N , Marth G , Abecasis G , Durbin R , 1000 Genome Project Data Processing Subgroup (2009) The sequence alignment/map format and SAMtools. Bioinformatics 25: 2078–2079 19505943 10.1093/bioinformatics/btp352 PMC2723002

[47] Quinlan AR , Hall IM (2010) BEDTools: a flexible suite of utilities for comparing genomic features. Bioinformatics 26: 841–842 20110278 10.1093/bioinformatics/btq033 PMC2832824

[48] Kent WJ , Zweig AS , Barber G , Hinrichs AS , Karolchik D (2010) BigWig and BigBed: enabling browsing of large distributed datasets. Bioinformatics 26: 2204–2207 20639541 10.1093/bioinformatics/btq351 PMC2922891

