# Supplementary material for: Clipped histone H3 is integrated into nucleosomes of DNA replication genes in the human malaria parasite Plasmodium falciparum
Source: EMBO Rep. 2019 Mar 4;20(4):e46331. doi: 10.15252/embr.201846331 (PMC6446197; doi:10.15252/embr.201846331)
Supplement: Supplementary file 1 — Appendix [file EMBR-20-e46331-s001.pdf]

Appendix for Herrera-Solorio *et al*, 2018

Table of Contents

Appendix Figure S1	Pg 2
Appendix Figure S2	Pg 3
Appendix Figure S3	Pg 4
Appendix Figure S4	Pg 5
Appendix Figure S5	Pg 8
Appendix Figure S6	Pg 10

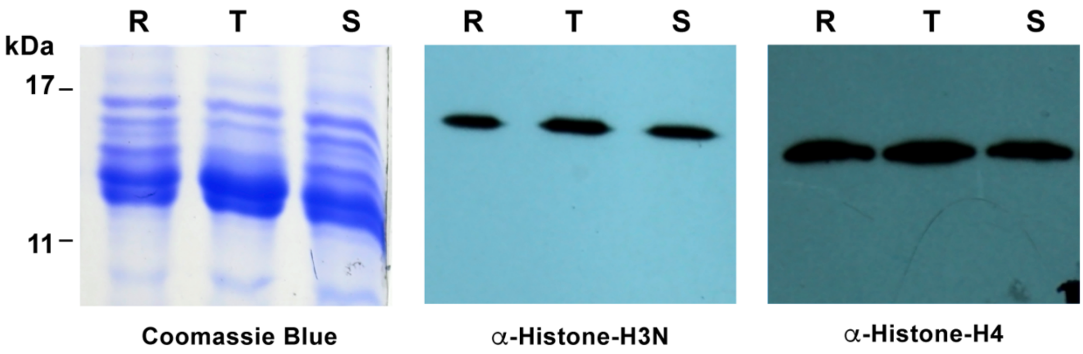

Appendix Figure S1: Mononucleosomes prepared from Ring (R), Trophozoite (T) and Schizont (S) stages of *P. falciparum* were separated on a denaturing polyacrylamide gel and visualized either by staining with Coomassie brilliant blue (C.B.) or by immunoblotting with anti-histone H3 N-terminal antibodies (α-Histone-H3n) or anti-Histone H4 antibodies. These data indicate that the clipping of Histone H3 occurs at the N-terminus.

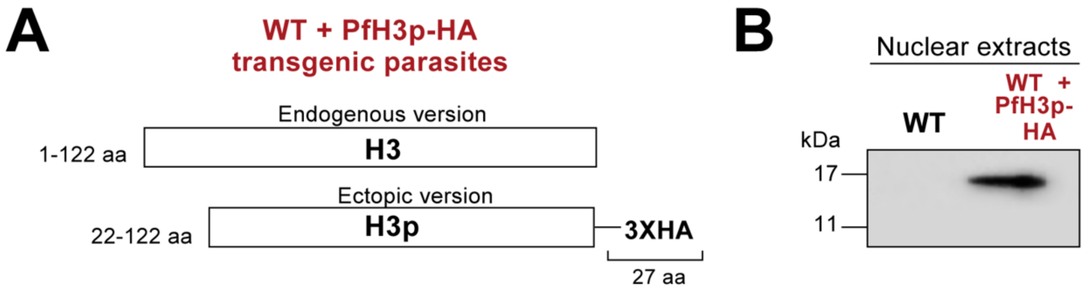

Appendix Figure S2: Ectopic expression of an HA-tagged version of clipped histone H3, PfH3p-HA **(A)**, was confirmed in parasite nuclear extracts using immunoblotting with anti-HA antibodies **(B)**.

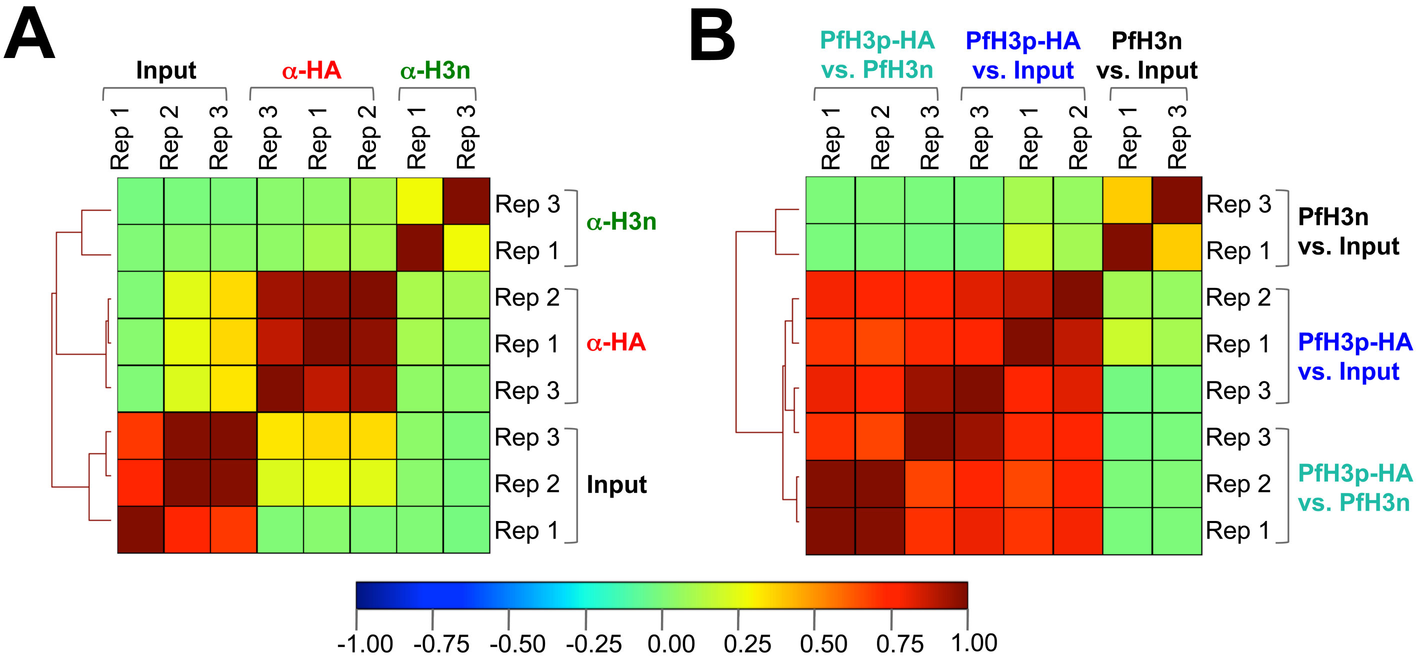
A
Input
α-HA
α-H3n
Rep 1
Rep 2
Rep 3
Rep 3
Rep 1
Rep 2
Rep 1
Rep 3
Rep 3
Rep 1
Rep 2
Rep 1
Rep 3
Rep 3
Rep 2
Rep 1
α-H3n
α-HA
Input
B
PfH3p-HA vs. PfH3n
PfH3p-HA vs. Input
PfH3n vs. Input
Rep 1
Rep 2
Rep 3
Rep 3
Rep 1
Rep 2
Rep 1
Rep 3
Rep 3
Rep 1
Rep 2
Rep 1
Rep 3
Rep 3
Rep 2
Rep 1
PfH3n vs. Input
PfH3p-HA vs. Input
PfH3p-HA vs. PfH3n
-1.00
-0.75
-0.50
-0.25
0.00
0.25
0.50
0.75
1.00

Appendix Figure S3: Correlations of **(A)** ChIP-seq bam files (genome-wide coverage normalized across 150 nt bins) and (**B**) fold enrichment of PfH3p-HA versus Input, PfH3p-HA versus PfH3n, or PfH3n versus Input comparisons for schizont stages (genome-wide coverage derived from MACS2, compared across contiguous 150 nt bins) were determined using a Pearson correlation analysis. The color scale indicates values of the Pearson correlation coefficient R from -1 to 1. Rep = Biological Replicate.

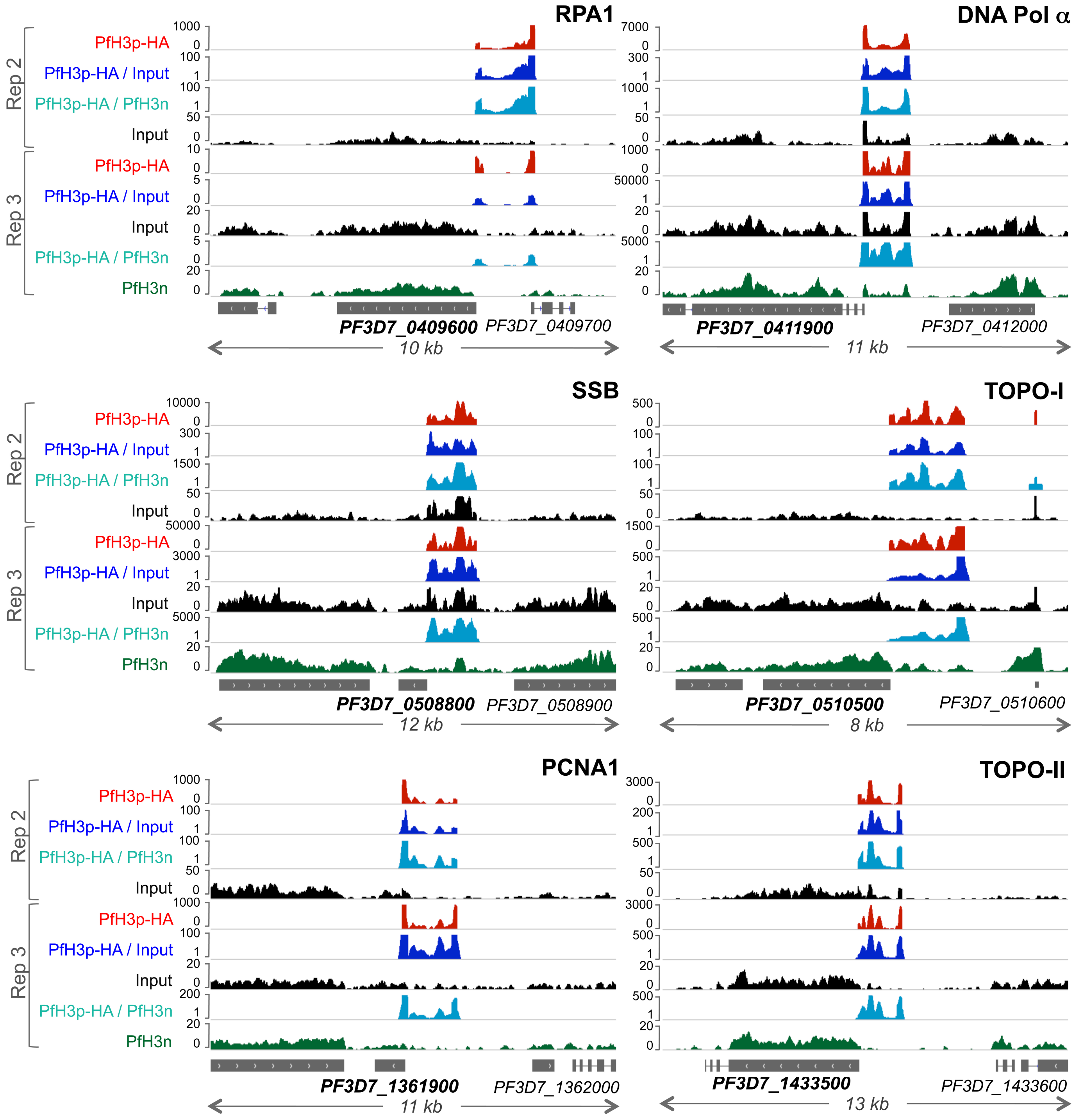

RPA1
DNA Pol α
SSB
TOPO-I
PCNA1
TOPO-II
Rep 2
Rep 3
PfH3p-HA
PfH3p-HA / Input
PfH3p-HA / PfH3n
Input
PfH3n
PF3D7_0409600
PF3D7_0409700
10 kb
PF3D7_0411900
PF3D7_0412000
11 kb
PF3D7_0508800
PF3D7_0508900
12 kb
PF3D7_0510500
PF3D7_0510600
8 kb
PF3D7_1361900
PF3D7_1362000
11 kb
PF3D7_1433500
PF3D7_1433600
13 kb

Appendix Figure S4: The genomic context of the six DNA replication genes enriched for PfH3p in schizont stages is shown for Biological Replicates 2 and 3. The fold enrichment of PfH3p-HA ChIP-seq signal over Input (PfH3p-HA / Input; blue) or PfH3n (PfH3p-HA / PfH3n; teal) is shown, as are the coverage plots of PfH3p-HA (red), PfH3n (green) and Input (black), which are represented as average reads per million over 1000 nt bins of the genome. DNA replication genes: Replication Protein A 1 (RPA1), Proliferating Cell Nuclear Antigen 1 (PCNA1), Single Stranded DNA binding protein (SSB), Topoisomerase I (TOPO-I), DNA polymerase alpha subunit (DNA pol a), and Topoisomerase II (TOPO-II) are highlighted and their directionality indicated.

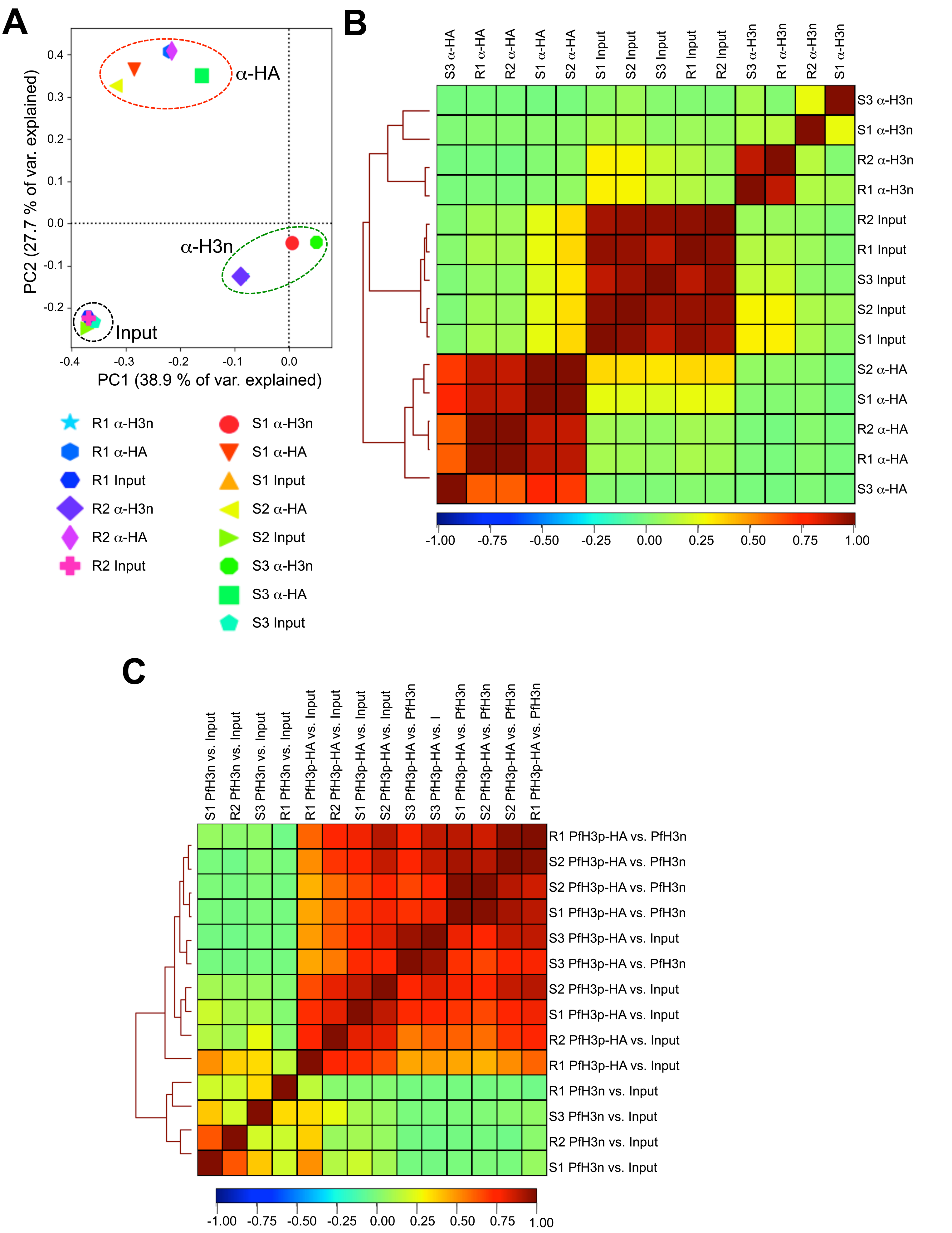
A
B
C
PC1 (38.9 % of var. explained)
PC2 (27.7 % of var. explained)
α-HA
α-H3n
Input
R1 α-H3n
R1 α-HA
R1 Input
R2 α-H3n
R2 α-HA
R2 Input
S1 α-H3n
S1 α-HA
S1 Input
S2 α-HA
S2 Input
S3 α-H3n
S3 α-HA
S3 Input
R1 PfH3p-HA vs. PfH3n
S2 PfH3p-HA vs. PfH3n
S1 PfH3p-HA vs. PfH3n
S3 PfH3p-HA vs. Input
S3 PfH3p-HA vs. PfH3n
S2 PfH3p-HA vs. Input
S1 PfH3p-HA vs. Input
R2 PfH3p-HA vs. Input
R1 PfH3p-HA vs. Input
R1 PfH3n vs. Input
S3 PfH3n vs. Input
R2 PfH3n vs. Input
S1 PfH3n vs. Input
S3 PfH3p-HA vs. I

Appendix Figure S5: Correlations between Ring and Schizont stage PfH3p genome-wide localization. A. Principal Component Analysis of the different ChIP-seq replicates (bigwig files derived from deduplicated bam files) for ring (R; two replicates R1 and R2) and schizont (S; three replicates S1, S2 and S3) stage samples was performed using the plotPCA function of deepTools on a multibigwig summary file, over 150 nt bins. The Eigen values of the top two principal components PC1 and PC2 are shown and meaningful clustering of replicates based on treatment is highlighted. Rep = Biological Replicate. **B.** Correlations of ChIP-seq bam files (genome-wide coverage normalized across 150 nt bins) were determined using a Pearson Correlation Analysis. The color scale indicates values of the Pearson correlation coefficient R from -1 to 1. **C.** Correlation of fold enrichment (genome-wide coverage derived from MACS2, compared across contiguous 150 nt bins) of PfH3p-HA versus Input, PfH3p-HA versus PfH3n, or PfH3n versus Input comparisons for ring or schizont stages was determined using a Pearson correlation analysis. The color scale indicates values of the Pearson correlation coefficient R from -1 to 1. The data indicate that the PfH3p enrichment between ring and schizont stages is highly correlated, and hence conserved.

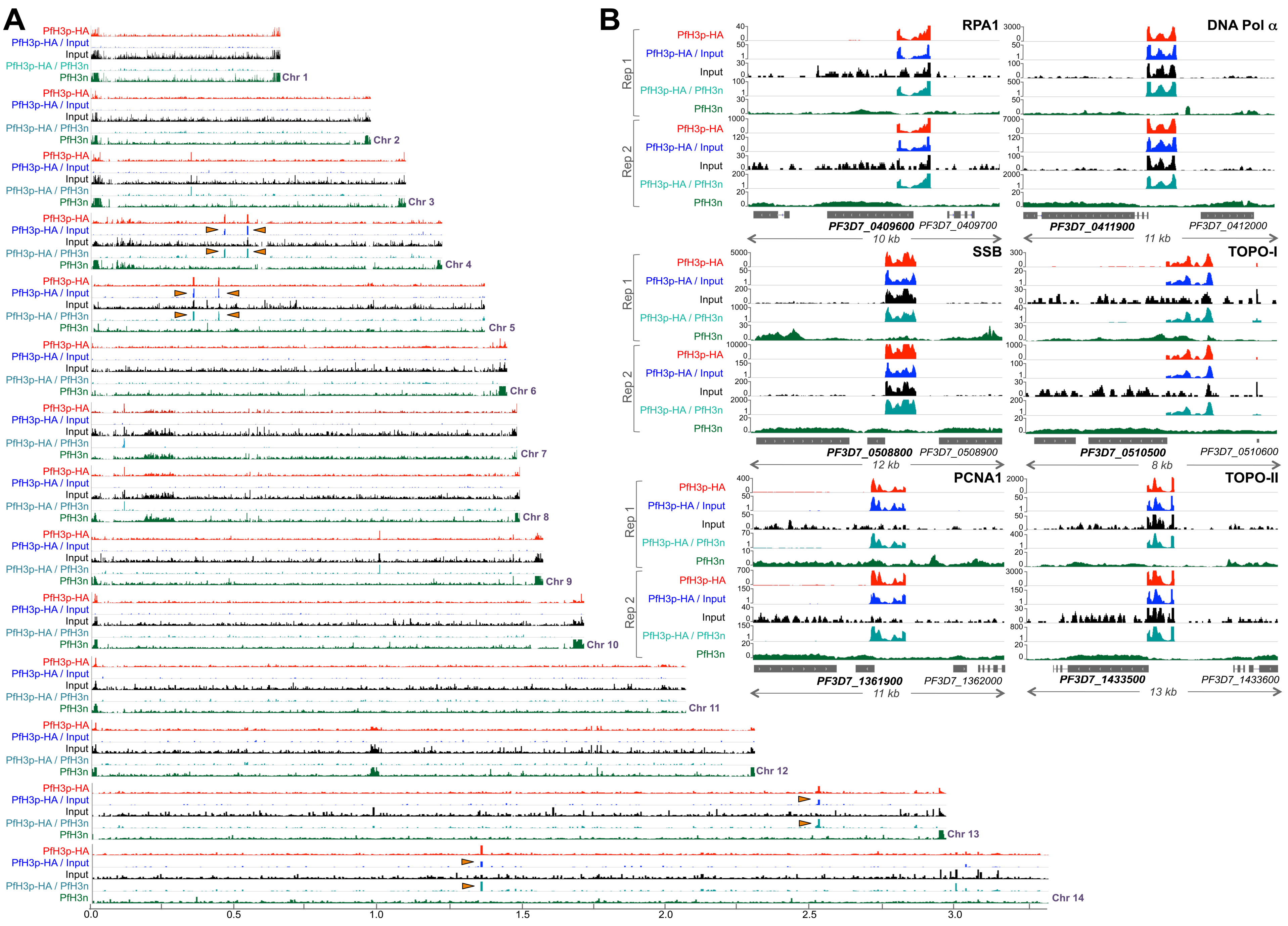

A
B
PfH3p-HA
PfH3p-HA / Input
Input
PfH3p-HA / PfH3n
PfH3n
Chr 1
Chr 2
Chr 3
Chr 4
Chr 5
Chr 6
Chr 7
Chr 8
Chr 9
Chr 10
Chr 11
Chr 12
Chr 13
Chr 14
0.0
0.5
1.0
1.5
2.0
2.5
3.0
Rep 1
Rep 2
RPA1
DNA Pol α
SSB
TOPO-I
PCNA1
TOPO-II
PF3D7_0409600
PF3D7_0409700
10 kb
PF3D7_0411900
PF3D7_0412000
11 kb
PF3D7_0508800
PF3D7_0508900
12 kb
PF3D7_0510500
PF3D7_0510600
8 kb
PF3D7_1361900
PF3D7_1362000
11 kb
PF3D7_1433500
PF3D7_1433600
13 kb

Appendix Figure S6: In *P. falciparum* Ring stages, ectopically expressed PfH3p is targeted to the 5' UTR of genes regulating DNA replication. A. Genome-wide distribution of ectopically expressed PfH3p-HA in *P. falciparum* ring stages is represented as fold-enrichment of PfH3p-HA ChIP-seq signal over Input (PfH3p-HA / Input in blue; y-axis scale 1-10) or over PfH3n (PfH3p-HA / Input in teal; y-axis scale 1-10) calculated using MACS2. The coverage of PfH3p-HA (red; y-axis scale 0-50), Input (black; y-axis scale 0-30) and PfH3n (green; y-axis scale 0-30) is also shown and represents average reads per million over 1000 nt bins of the genome. The x-axis represents chromosome size in Mb. Major MACS2-derived peaks identified in all replicates are indicated using an orange arrowhead. Note that the data correspond to Biological Replicate 1. **B.** The genomic context of the six peaks identified in both PfH3p-HA ChIP-seq replicates for Ring stages is shown. The fold enrichment of PfH3p-HA ChIP-seq signal over Input (PfH3p-HA / Input; blue) or PfH3n (PfH3p-HA / PfH3n; teal) is shown, as are the coverage plots of PfH3p-HA (red), PfH3n (green) and Input (black), which are represented as average reads per million over 1000 nt bins of the genome. DNA replication genes: Replication Protein A 1 (RPA1), Proliferating Cell Nuclear Antigen 1 (PCNA1), Single Stranded DNA binding protein (SSB), Topoisomerase I (TOPO-I), DNA polymerase alpha subunit (DNA pol a), and Topoisomerase II (TOPO-II) are highlighted and their directionality indicated. Rep = Biological Replicate.
