# Supplementary figures and images for: Clipped histone H3 is integrated into nucleosomes of DNA replication genes in the human malaria parasite Plasmodium falciparum
Source: EMBO Rep. 2019 Mar 4;20(4):e46331. doi: 10.15252/embr.201846331 (PMC6446197; doi:10.15252/embr.201846331)

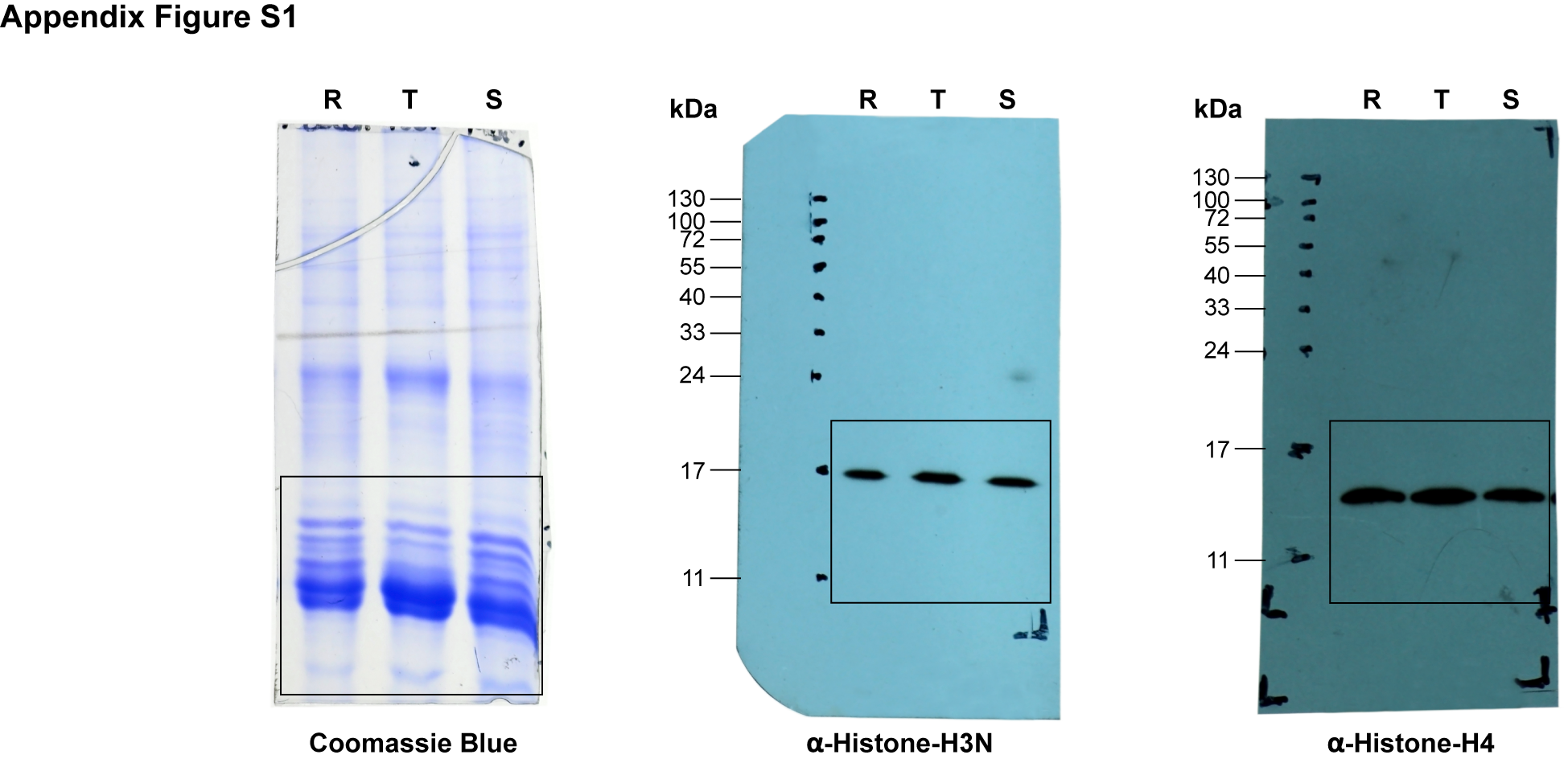

Supplement: Supplementary file 9 — Source Data for Appendix [file EMBR-20-e46331-s012.zip › Appendix_source_data/Fig_S1_source_data.tif]

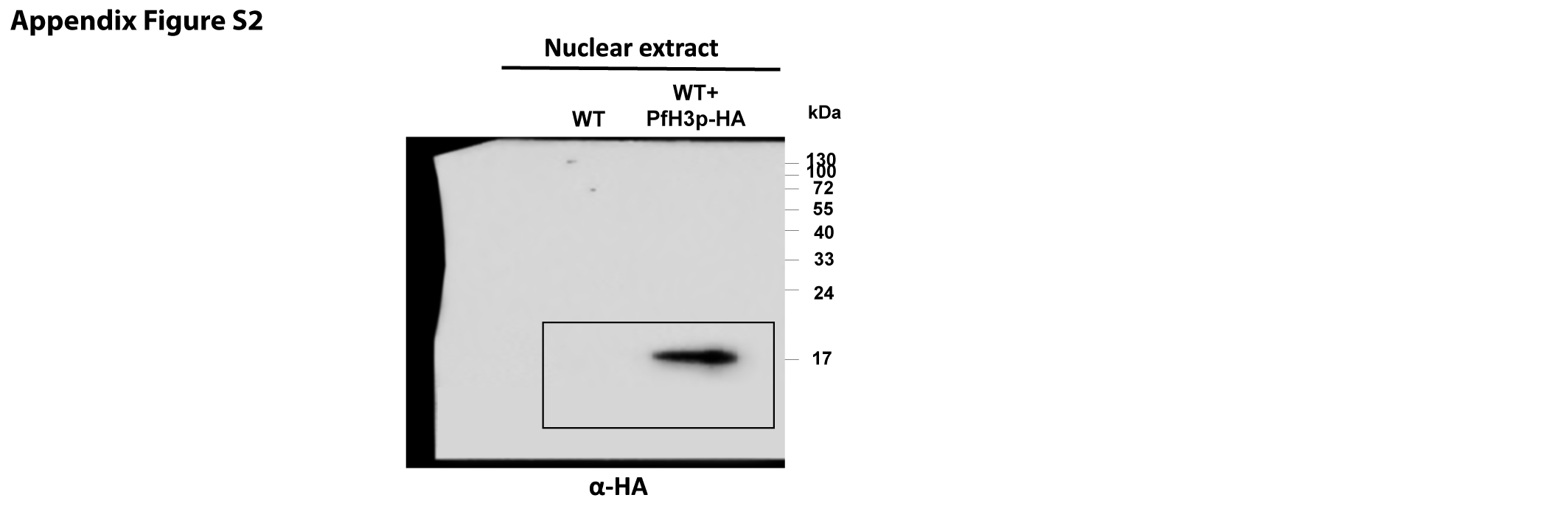

Supplement: Supplementary file 9 — Source Data for Appendix [file EMBR-20-e46331-s012.zip › Appendix_source_data/Fig_S2_source_data.tif]

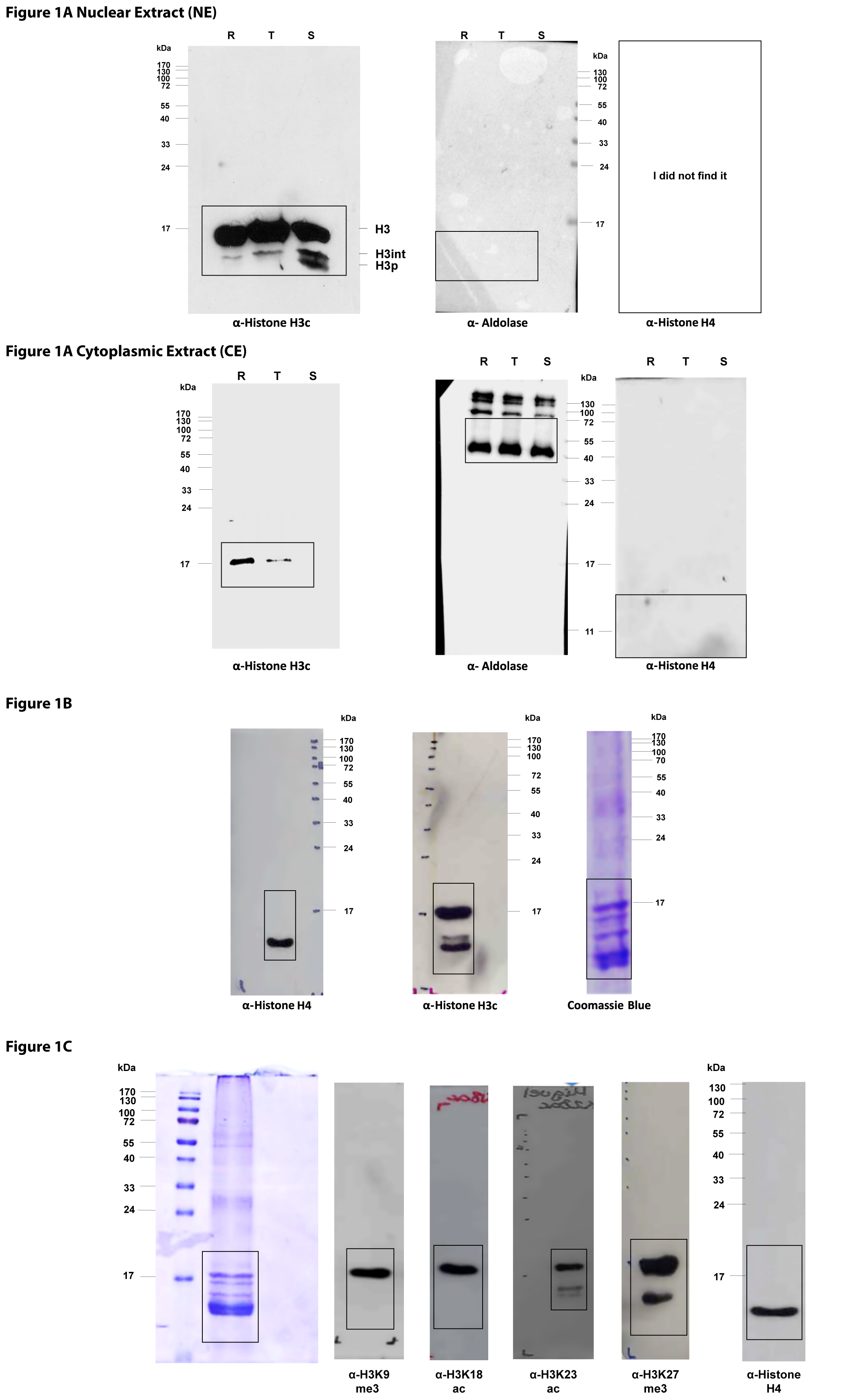

Supplement: Supplementary file 11 — Source Data for Figure 1 [file EMBR-20-e46331-s009.tif]

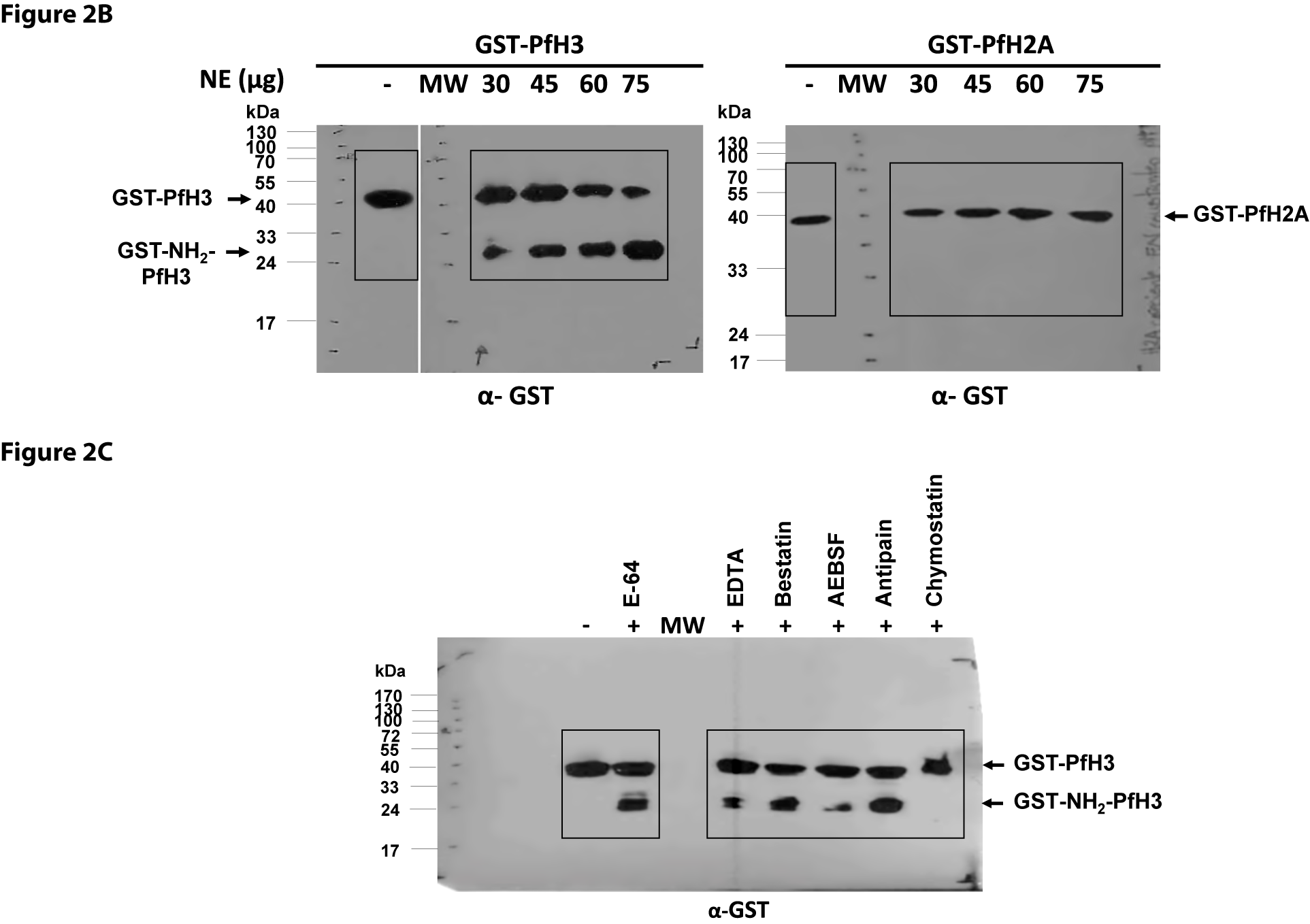

Supplement: Supplementary file 12 — Source Data for Figure 2 [file EMBR-20-e46331-s010.tif]

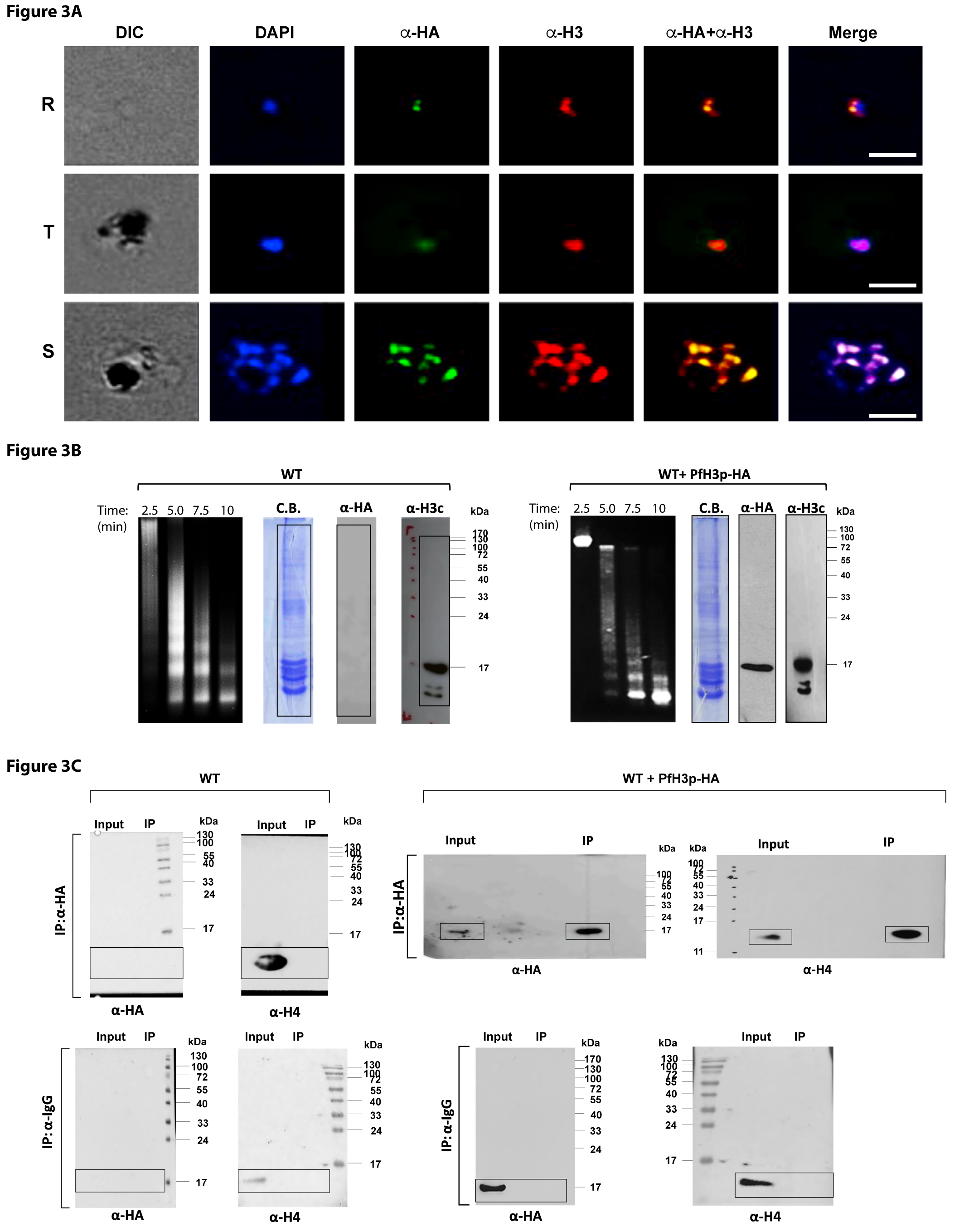

Supplement: Supplementary file 13 — Source Data for Figure 3 [file EMBR-20-e46331-s011.tif]
